# Potent GH20 *N*-Acetyl-β-d-hexosaminidase Inhibitors: *N*-Substituted 3-acetamido-4-amino-5-hydroxymethyl-cyclopentanediols

**DOI:** 10.3390/molecules23030708

**Published:** 2018-03-20

**Authors:** Patrick Weber, Seyed A. Nasseri, Bettina M. Pabst, Ana Torvisco, Philipp Müller, Eduard Paschke, Marion Tschernutter, Werner Windischhofer, Stephen G. Withers, Tanja M. Wrodnigg, Arnold E. Stütz

**Affiliations:** 1 Glycogroup, Institute of Organic Chemistry, Graz University of Technology, Stremayrgasse 9, A-8010 Graz, Austria; patrick.weber@tugraz.at (P.W.); t.wrodnigg@tugraz.at (T.M.W.); 2 Chemistry Department, University of British Columbia, 2036 Main Mall, Vancouver, BC V6T 1Z1, Canada; snasseri@chem.ubc.ca (S.A.N.); withers@chem.ubc.ca (S.G.W.); 3 Laboratory of Metabolic Diseases, Department of Pediatrics, MedUni Graz, Auenbruggerplatz 30, A-8036 Graz, Austria; bettina.pabst@medunigraz.at (B.M.P.); eduard.paschke@inode.at (E.P.); marion.tschernutter@medunigraz.at (M.T.); werner.windischhofer@medunigraz.at (W.W.); 4 Institute of Inorganic Chemistry, Graz University of Technology, Stremayrgasse 9, A-8010 Graz, Austria; ana.torviscogomez@tugraz.at (A.T.); philipp.mueller@tugraz.at (P.M.)

**Keywords:** aminocyclopentane, *N*-acetyl-d-hexosaminidase inhibitor, Tay-Sachs

## Abstract

From 1,2;3,4-di-*O*-isopropylidene-d-galactopyranose, a preliminary series of highly functionalized amino(hydroxymethyl)cyclopentanes was easily available. These amine-containing basic carbasugars featuring the d-*galacto* configuration are potent inhibitors of the GH20 β-d-hexosaminidases probed and may bear potential as regulators of *N*-acetyl-d-hexosaminidase activities in vivo.

## 1. Introduction

In humans, *N*-acetyl-d-hexosaminidases (HexA, HexB, and HexS) play essential roles in the lysosomal processing of degradation-bound glycolipids as well as glycans [1]. *O*-GlcNAcase [2] removes O-linked GlcNAc residues from serine or threonine in nucleocytoplasmic proteins. Another *N*-acetylhexosaminidase, HexD, was recently reported and has been mentioned in context with rheumatoid arthritis [3].

Whereas the retaining lysosomal *N*-acetyl-α-d-hexosaminidases of GH89 follow the standard double-displacement mechanism, *N*-acetyl-β-d-hexosaminidases of GH20, as well as the GH84 *O*-GlcNAcase, GH18 chitinases, and GH85 *endo*-*N*-acetyl-β-d-glucosaminidases exploit anchimeric assistance by the sugar’s *N*-acetyl group, which upon intramolecular attack of the intermediary oxocarbenium ion forms an α-configured oxazoline intermediate. This, in turn, is attacked from the β-face by an activated water molecule [4]. Interesting reviews on hexosaminidases are available [5,6].

Potent and highly selective inhibitors are required for studying these enzymes and their physiological significance. In particular, potential therapeutic applications in context with lysosomal disorders [7], cancer [8], and Alzheimer’s disease [9] require high degrees of selectivity for any of the enzymes mentioned above over the other respective *N*-acetylhexosaminidases.

**Figure 1:**
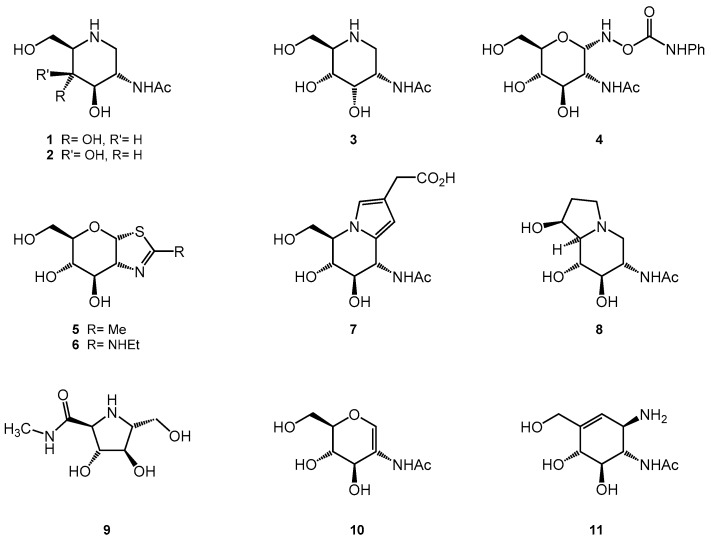
Examples of established as well as recently reported *N*-acetyl-d-hexosaminidase inhibitors.

Amongst *N*-acetylhexosaminidase inhibitors (Figure 1), 2-acetamido-1,2-dideoxynojirimycin, **1** [10] and diastereomers such as the d-*galacto* (**2**) [11] and the d-*allo* (**3**) [12] analogs, PUGNAc (**4**) [13], and NAG-thiazoline (NGT, **5**) [14] have attracted considerable attention. Furthermore, Thiamet G (**6**) [15], nagstatin (**7**) [16], and 6-acetamido-6-deoxycastanospermine (**8**) [17], various pyrrolidine derivatives (for example, compound **9** [18]), as well as 2-*N*-acetyl glycals including **10** [19] have been reported. Amongst carbacyclic hexosaminidase inhibitors, pyranoid carbasugar acetamidodeoxy-β-valienamine (**11**) has to be mentioned [20]. These inhibitors are either substrate/product analogs or, in the case of bicyclic systems such as NGT, are chemically stable structural analogues of the above-mentioned intermediate generated by anchimeric assistance of the *N*-acetyl group at C-1 at the first transition state of enzymatic *N*-acetylhexosaminide hydrolysis.

Jäger and co-workers [21] have directed our attention toward cyclopentanoid basic sugar analogs as potentially useful inhibitors of lysosomal glycosidases. Relying on the pioneering synthetic work by Vasella and co-workers [22] based on guiding contributions by Padwa [23] as well as Oppolzer [24], we had thus investigated cyclopentane-based β-galactosidase inhibitors [25] and have recently extended our range of new compounds by addition of *N*-acetyl-β-d-galactosaminide and -β-d-glucosaminide analogs.

## 2. Results and Discussion

### 2.1. Synthesis

**Scheme 1:**
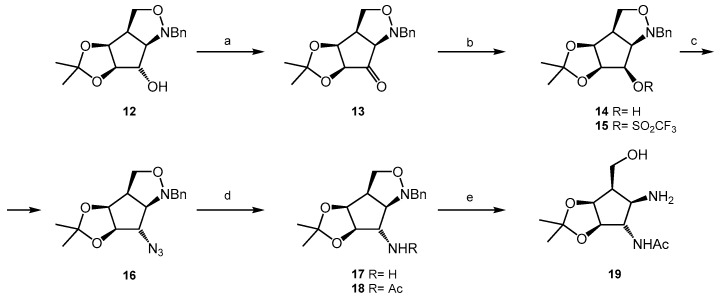
Introduction of the acetamido group by double inversion approach. a: Swern or Dess-Martin; b: NaBH_4_, MeOH; c: (1) Tf_2_O, pyr., (2) NaN_3_, DMF; d: (1) Zn/NH_4_Cl, (2) Ac_2_O, pyr.; e: Pd(OH)_2_/C, H_2_.

Starting from known [26] *N*-benzylisoxazolidine **12** (Scheme 1), by a simple oxidation/reduction sequence, ketone **13** provided epimer **14** in high yield.

**Figure 2:**
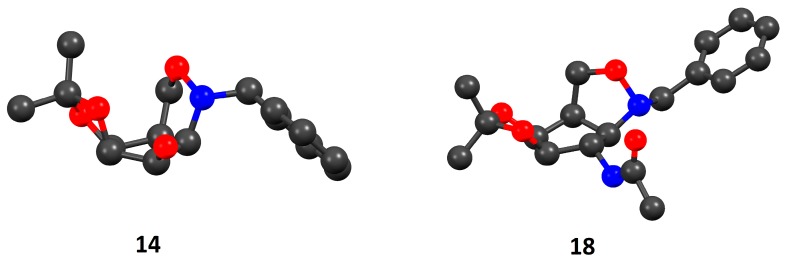
Crystal stuctures of alcohol **14** (CCDC 1826202) and acetamide **18** (CCDC 1826203).

Its structural identity can unambiguously be verified by X-ray structure determination (Figure 2). The corresponding triflate **15** provided azidodeoxy derivative **16** by clean inversion of configuration. Intermediate **16**, upon reduction with Zn under slightly acidic conditions and subsequent conventional *N*-acetylation, highly selectively furnished, via free amine **17**, desired acetamido compound **18**, which yielded crystals of sufficient quality for XRD (Figure 2).

**Scheme 2:**
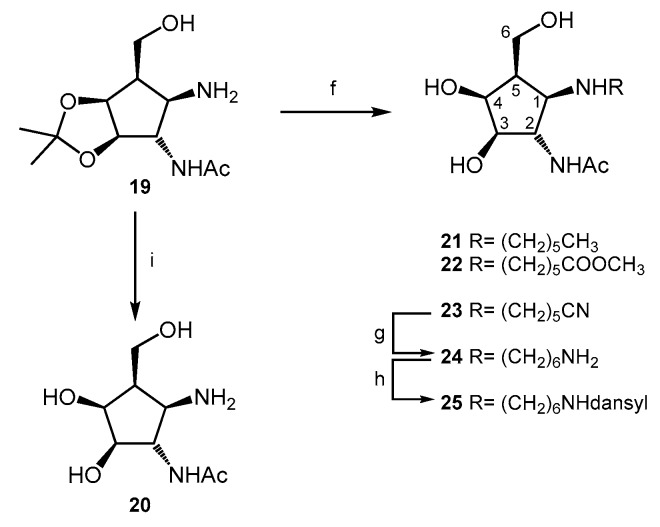
Conversion of crucial intermediate **19** into free inhibitor **20** and *N*-alkyl derivatives thereof. f: (1) resp. halogenoalkane, NaHCO_3_, DMF, (2) HCl/MeOH; g: Raney-Ni, H_2_, MeOH; h: dansyl chloride, NaHCO_3_, MeOH; i: HCl/MeOH. (Structures show the numbering system applied for NMR-analysis for easier comparison with other d-galactosaminide related compounds).

From tricycle **18**, by hydrogenolysis over Perlman’s catalyst, cyclopentane **19** featuring a free amine was obtained in good yield. Subsequent acidic deprotection yielded free aminotriol **20** (Scheme 2).

Chemoselective *N*-alkylation of intermediate **19** provided the corresponding *N*-hexyl (**21**), *N*-methoxycarbonylpentyl (**22**), as well as *N*-cyanopentyl (**23**) derivatives. By reduction of the nitrile function in **23**, primary amine **24** became available, which was converted into fluorescent dansylaminohexyl derivative **25** with the aid of dansyl chloride.

### 2.2. Biological Evaluation

New compounds turned out particularly potent inhibitors of *Streptomyces plicatus N*-acetyl-β-hexosaminidase (SpHex) with *K*_i_-values in the sub-nanomolar range (Table 1). By introduction of a dansylamido moiety into the alkyl chain, this activity was further improved as shown by the 60 pM value determined for inhibitor **25** when compared to analogue **21**. With the same enzyme, compound **1** exhibited *K*_i_ = 80 μM [26].

The latter two compounds were also screened with Tay-Sachs disease-related human lysosomal *N*-acetyl-β-hexosaminidase A. For comparison, pyrimethamine [27], which under the same screening conditions exhibited IC_50_ = 62 μM, as well as 2-acetamido-1,2-dideoxynojirimycin,**1** (IC_50_ 31 μM) [28] were included in these studies.

Inhibitors **21** and **25** exhibited excellent properties with this vital human enzyme, considerably exceeding the activities of the reference compounds probed in this study.

In conclusion, compounds of this new aminocyclopentane-derived family of *N*-acetylgalactosaminide mimetics represent a potentially interesting class of *N*-acetyl-d-hexosaminidase inhibitors and pharmacological chaperones for treatment of Tay Sachs disease, in particular, when also considering their simplicity of synthesis.

## 3. Materials and Methods

### 3.1. General Methods

Optical rotations were measured at 20 °C on a Perkin Elmer (Waltham, MA, USA) 341 polarimeter at a wave length of 589 nm and a path length of 10 cm.

NMR spectra were recorded on a Varian (Palo Alto, CA, USA) INOVA 500 operating at 499.82 MHz (^1^H), and at 125.894 MHz (^13^C), or on a Bruker (Billerica, MA, USA) Ultrashield spectrometer at 300.36 and 75.53 MHz, respectively. CDCl_3_ was employed for protected compounds and CD_3_OD as well as D_2_O for unprotected inhibitors. Chemical shifts are listed in delta employing residual, non-deuterated solvent as the internal standard. Signals were assigned unambiguously by COSY and HSQC analysis. The signals of the *N*-dansyl group are located in the expected regions and are not listed explicitly. For easier comparison with other *N*-acetylgalactosaminide analogues, interpretation of NMR-spectra was performed according to the carbohydrate-related numbering system depicted in Scheme 2. MALDI-TOF and EI-TOF mass spectrometry were performed on a Micromass (Waters Corporation, Milford, MA, USA) TofSpec 2E Time-of-Flight mass spectrometer. Analytical TLC was performed on pre-coated aluminum plates silica gel 60 F254 (E. Merck, Darmstadt, Germany 5554) and detected with UV light (254 nm). For staining, a solution of vanillin (9 g) in a mixture of H_2_O (950 mL)/EtOH (750 mL)/H_2_SO_4_ (120 mL) or ceric ammonium molybdate (100 g ammonium molybdate/8 g ceric sulfate in 1 l 10% H_2_SO_4_) were employed, followed by heating on a hotplate. For column chromatography, silica gel 60 (230–400 mesh, E. Merck 9385) or silica gel 60 (Acros Organics (Thermo Fisher Scientific Inc., Waltham, MA, USA), AC 24036) were used. CCDC contains the supplementary crystallographic data for this paper. These data can be obtained free of charge via http://www.ccdc.cam.ac.uk/conts/retrieving.html (or from the CCDC, 12 Union Road, Cambridge CB2 1EZ, UK; Fax: +44 1223 336033; E-mail: deposit@ccdc.cam.ac.uk).

### 3.2. Biochemical Methods

*Streptomyces plicatus N*-acetyl-β-hexosaminidase, SpHex, was expressed and purified in *E. coli* as described previously [27]. Kinetic studies were performed at 25 °C in the assay buffer (sodium phosphate (50 mM), sodium citrate (50 mM), NaCl (100 mM), BSA (2 mg/mL), pH = 6.0). The enzyme was incubated with different concentrations of the inhibitors for 2–5 min prior to the start of the reaction by addition of the substrate (4-nitrophenyl *N*-acetyl-β-d-glucosaminide) and the initial rates were measured by monitoring the increase in absorbance at 405 nm for three to five minutes using a microplate reader (Synergy H1, BioTek, VT, USA). *K*_i_ determinations were performed using two or three different substrate concentrations. For each one of these substrate concentrations a range of five to eight different inhibitor concentrations bracketing the ultimately determined *K*_i_ value were used. Dixon plots (1/rate vs [I]) were constructed to validate the use of a competitive inhibition model. The data were then fit to a competitive inhibition model using non-linear regression analysis with GraFit 7.0 (Erithacus Software, UK). Assays were done twice using enzyme concentrations of 0.3 nM and 0.03 nM respectively in order to check compliance with the assumptions of Michaelis Menten kinetics ([E] << [I]). In addition, for compound **25**, the assays were done a third time with the concentration of enzyme lowered to 0.003 nM. In all cases, the *K*_i_ values were in good agreement.

Human skin fibroblasts (wild type) were grown in minimal essential medium (MEM) with Earle’s Salts (Sigma Aldrich, St. Louis, MO, USA) containing 10% fetal bovine serum, 400 µM l-glutamine, and 50 µg/mL gentamycin at 37 °C and 5% CO_2_. All cells used in this study were between the third and nineteenth passages.

All inhibitors were dissolved in DMSO in a concentration of 10 mM and diluted in 10 mM phosphate buffer (pH 7.0) containing 100 mM NaCl, 0.01% NaN_3_, and 0.01% Triton for the IC_50_-measurements.

Human *N*-acetyl-β-hexosaminidase A activity measurements were performed in duplicate assays unless otherwise stated. Fibroblast cells of three single 80 cm^2^ flasks were harvested by trypsinization in 500 µL (each of them) 0.9% NaCl containing 0.01% Triton, homogenized by sonication (4 times 15 s, Bandelin (Berlin, Germany) Sonopuls ultrasound homogenator mini 20) and centrifuged at 13.000 rpm for 1 min in a table top centrifuge (Biofuge Pico, Heraeus, Hanau, Germany). Protein amounts were determined according to the method of Lowry. For assessment of *N*-acetyl-β-hexosaminidase A activity, 10 µL (diluted 1:10) of cell homogenate were mixed with 90 µL 0.9% NaCl and 200 µL of 1 mM (4-methyl)umbelliferyl *N*-acetyl-β-glucosaminide-6-sulfate. Na (Glycosynth, Warrington, UK) in McIlvains phosphate/citrate puffer, pH 4,4. After incubation at 37 °C for 60 min, the reaction was stopped by adding 2.5 mL 400 mM glycine/NaOH (pH 10.4). The amount of hydrolyzed 4-methylumbelliferone was determined with a fluorescence spectrometer (F7000 Hitachi, Chiyoda, Japan).

Modified β-hexosaminidase A assays were used to estimate the half maximal inhibitory concentration (IC_50_) of the particular inhibitor. For IC_50_ determination, 0.001 to 100 µM of inhibitor was added to the assay mixture.

Activity was measured in normal fibroblasts. Data analysis was performed with Microcal™ Origin^®^ v6.0 (Origin Lab, Northampton, MA, USA) using a non-linear curve fitting module based on sigmoid curve fitting.

### 3.3. (3aR,3bS,6aR,7R,7aR)-Hexahydro-5,5-dimethyl-1-phenyl-1H-[1,3]dioxolo[3,4]cyclopent[1,2-c]isoxazol-7-ol or 1-l-(1,2,3,4,5)-1^1^,2^1^-Anhydro-1-hydroxymethyl-2-(N-hydroxy)benzylamino-4,5-O-isopropylidene-3,4,5-cyclopentanetriol **14**

(a) *Via Swern oxidation*: To a solution of oxalyl chloride (1.12 mL, 13.0 mmol) in CH_2_Cl_2_ (20 mL), DMSO (1.11 mL, 15.7 mmol) was added dropwise at −60 °C. After 15 min, a 50% solution (*w*/*v*) of alcohol **12** (1.52 g, 5.22 mmol) in CH_2_Cl_2_ was added and the reaction was stirred for 15 min when Et_3_N (2.89 mL, 20.9 mmol) was added. The reaction mixture was allowed to reach ambient temperature and methanol (50 mL) and NaBH_4_ (0.39 g, 10.4 mmol) were added. When completed conversion of cyclopentanone **13** was observed (tlc, 30 min), solvents were removed under reduced pressure and crude alcohol **14** was dissolved in CH_2_Cl_2_. The organic layer was extracted with saturated aqueous NaHCO_3_, dried (Na_2_SO_4_), filtered and concentrated under reduced pressure. Purification on silica gel (cyclohexane/ethyl acetate 3:1 *v*/*v*) provided compound **14** as a pale yellow syrup (848 mg, 2.91 mmol, 55.8% from epimer **12**).

(b) *Via Dess-Martin oxidation*: A 10% solution (*w*/*v*) of alcohol **12** (1.05 g, 3.60 mmol) in CH_2_Cl_2_ was stirred with Dess-Martin periodinane (1.68 g, 3.96 mmol) at ambient temperature for 10 min. After completed conversion, the reaction mixture was washed with saturated aqueous NaHCO_3_, dried (Na_2_SO_4_), and filtered. Removal of solvents under reduced pressure gave the crude product **13**.

To a solution of crude ketone **13** in methanol (20 mL), NaBH_4_ (0.273 g, 7.21 mmol) was added. When completed conversion was detected (tlc, 30 min), solvents were removed under reduced pressure, and the crude product was dissolved in CH_2_Cl_2_. The organic layer was extracted with saturated aqueous NaHCO_3_, dried (Na_2_SO_4_), filtered, and concentrated under reduced pressure. Purification on silica gel (cyclohexane/ethyl acetate 3:1 *v*/*v*) provided compound **14** as a pale yellow syrup (0.769 g, 2.64 mmol, 73.2% over two steps).

After extended storage, a compound sample provided a few minute crystals, one of which could be exploited for X-ray structure determination (CCDC 1826202).

${\left[\alpha \right]}_{D}^{20}$: +44.6 (*c* = 0.86, CHCl_3_); ^1^H-NMR (300 MHz, CDCl_3_) δ = 7.42–7.23 (m, 5H, aromatic NBn), 4.58 (dd, 1H, *J*_2,3_ = *J*_3,4_ = 5.5 Hz, H-3), 4.50 (dd, 1H, *J*_4,5_ = 7.5 Hz, H-4), 4.33 (d, 1H, *J*_5,6a_ < 1 Hz, *J*_6a,6b_ = 8.7 Hz, H-6a) 4.12 (d, 1H, *J* = 13.3 Hz, N-CH_2_-Ph), 4.04 (m, 1H, H-2), 3.97 (dd, 1H, *J*_5,6b_ = 6.4 Hz, H-6b), 3.91 (dd, 1H, N-CH_2_-Ph), 3.68 (dd, 1H, *J*_1,2_ = 7.6 Hz, H-1), 3.48 (bs, 1H, 6-OH), 3.07 (ddd, 1H, H-5), 1.57, 1.25 (2s, 3H each, C(CH_3_)_2_). ^13^C-NMR (75.5 MHz, CDCl_3_): δ = 137.2 (ipso NBn), 129.2, 128.6, 127.6 (aromatic NBn), 112.8 (C(CH_3_)_2_), 81.7 (C-3), 78.3 (C-4), 73.2 (C-1), 71.2 (C-2), 65.6 (C-6), 62.1 (N-CH_2_-Ph), 49.6 (C-5), 25.4, 25.0 (C(CH_3_)_2_).

MS (MALDI): Calcd for [C_16_H_21_NO_4_Na]: *m/z* 314.1368 [M + Na]^+^; Found [M + Na]^+^ 314.1368.

### 3.4. (3aR,3bS,6aR,7S,7aR)-Hexahydro-7-azido-5,5-dimethyl-1-phenyl-1H-[1,3]dioxolo[3,4]cyclopent[1,2-c]isoxazol or 1-l-(1,2,4,5/3)-1^1^,2^1^-Anhydro-3-azido-1-hydroxymethyl-2-(N-hydroxy)benzylamino-4,5-O-isopropylidene-4,5-cyclopentanediol **16**

A solution of alcohol **14** (848 mg, 2.91 mmol) in CH_2_Cl_2_ (20 mL) was cooled to 0 °C. Pyridine (0.940 mL, 11.6 mmol) and trifluoromethanesulfonyl anhydride (0.637 mL, 3.78 mmol) were added. When completed conversion of the starting material was observed (10 min), the reaction mixture was washed consecutively with HCl (6%) and saturated aqueous NaHCO_3_. After drying with Na_2_SO_4_, the suspension was filtered, and the solvent was removed at room temperature under reduced pressure. Resulting crude triflate **15** was dissolved in DMF (20 mL), NaN_3_ (1.14 g, 17.5 mmol) was added and the mixture was stirred at ambient temperature for 60 min. The reaction mixture was then concentrated under reduced pressure, the residue was dissolved with CH_2_Cl_2_, and the solution was washed with brine. The organic layer was dried (Na_2_SO_4_), filtered, and concentrated under reduced pressure. Purification of the remaining residue on silica gel (cyclohexane/ethyl acetate 10:1 *v*/*v*) provided azidodeoxy compound **16** (568 mg, 1.80 mmol, 61.7% from **14**). ${\left[\alpha \right]}_{D}^{20}$: +74.8 (*c* = 1.09, CHCl_3_); ^1^H-NMR (300 MHz, CDCl_3_) δ = 7.44–7.23 (m, 5H, aromatic NBn), 4.59 (dd, 1H, *J*_3,4_ = *J*_4,5_ = 6.9 Hz, H-4), 4.31 (dd, 1H, *J*_5,6a_ = 3.5 Hz, *J*_6a,6b_ = 8.9 Hz, H-6a), 4.28 (m, 1H, H-3), 4.07 (d, 1H, H-6b), 4.01 (d, 1H, *J* = 12.6 Hz, N-CH_2_-Ph), 3.78 (dd, 1H, *J*_1,2_ = *J*_2,3_ = 7.5 Hz, H-2), 3.70 (d, 1H, N-CH_2_-Ph), 3.61 (dd, 1H, *J*_1,5_ = 8.2 Hz, H-1), 3.29 (dddd, 1H, H-5), 1.54, 1.29 (2s, 3H each, C(CH_3_)_2_. ^13^C-NMR (75.5 MHz, CDCl_3_): δ = 136.5 (ipso NBn), 129.1, 128.6, 127.8 (aromatic NBn), 113.4 (C(CH_3_)_2_), 84.5 (C-3), 77.4 (C-4), 75.6 (C-1), 69.9 (C-2), 64.9 (C-6), 59.5 (N-CH_2__-_Ph), 45.6 (C-5), 27.7, 25.7 (C(CH_3_)_2_).

MS (EI): Calcd for [C_16_H_20_N_4_O_3_]: *m*/*z* 316.1535 [M]^+^; Found [M]^+^ 316.1532.

### 3.5. (3aR,3bS,6aR,7S,7aR)-Hexahydro-7-acetamido-5,5-dimethyl-1-phenyl-1H-[1,3]dioxolo[3,4]cyclopent[1,2-c]isoxazol or 1-l-(1,2,4,5/3)-1^1^,2^1^-Anhydro-3-acetamido-1-hydroxymethyl-2-(N-hydroxy)benzylamino-4,5-O-isopropylidene-4,5-cyclopentanediol **18**

To a stirred suspension of zinc (1.17 g, 18.0 mmol) and NH_4_Cl (0.961 g, 18.0 mmol) in methanol (20 mL) a 50% solution (*w*/*v*) of azidocyclopentane **16** (848 mg, 2.91 mmol) in methanol was added. After completed conversion of the starting material (2 h), the mixture was filtered and concentrated under reduced pressure. The resulting crude amine **17** was dissolved in pyridine (20 mmol) and treated with acetic anhydride (0,255 mL, 2.69 mmol) and 4-DMAP (5 mg) at 0 °C. After completed consumption of amine **17**, the reaction was quenched with methanol, and the solvents were removed under reduced pressure. The residue was dissolved in CH_2_Cl_2_ and consecutively washed with HCl (6%) and saturated aqueous NaHCO_3_, dried (Na_2_SO_4_) and filtered. Purification over silica gel chromatography (cyclohexane/ethyl acetate 2:1 *v*/*v*) provided acetamide **18** (422 mg, 1.27 mmol, 70.7% from **16**) as a pale yellow syrup. ${\left[\alpha \right]}_{D}^{20}$: +11.1 (*c* = 0.82, CHCl_3_); ^1^H-NMR (300 MHz, CDCl_3_) δ = 7.38–7.23 (m, 5H, aromatic NBn), 6.11 (d, 1H, NHCOCH_3_), 4.95 (dd, 1H, *J*_2,3_ = *J*_3,4_ = 6.3 Hz, H-3), 4.75 (dd, 1H, *J*_4,5_ = 7.1 Hz, H-4), 4.29 (dd, 1H, *J*_5,6a_ = 4.1 Hz, *J*_6a,6b_ = 8.7 Hz, H-6a), 4.22 (dd, 1H, *J*_1,2_ = *J*_1,5_ = 8.0 Hz, H-1), 4.06 (dd, 1H, *J*_5,6b_ = 8.9 Hz, H-6b), 3.98 (d, 1H, *J* = 12.9 Hz, N-CH_2_-Ph), 3.67 (d, 1H, N-CH_2_-Ph), 3.42 (m, 1H, H-5), 3.34 (dd, 1H, H-2), 1.83 (s, 3H, NHCOCH_3_), 1.51, 1.29 (2s, 3H each, C(CH_3_)_2_). ^13^C-NMR (75.5 MHz, CDCl_3_): δ = 170.7 (NHCOCH_3_), 137.0 (ipso NBn), 129.2, 128.5, 127.6 (aromatic NBn), 112.6 (C(CH_3_)_2_), 83.2 (C-3), 78.4 (C-4), 74.3 (C-1), 65.4 (C-6), 63.9 (C-2), 59.9 (N-CH_2_-Ph), 47.1 (C-5), 27.3, 25.4 (C(CH_3_)_2_), 23.6 (NHCOCH_3_).

After extended storage, a compound sample provided small crystals which could be employed for X-ray structure determination (CCDC 1826203).

MS (EI): Calc for [C_18_H_24_N_2_O_4_]: *m/z* 332.1736 [M]^+^; Found [M]^+^ 332.1737.

### 3.6. (3aS,4R,5R,6S,6aR)-5-Amino-tetrahydro-6-acetamido-2,2-dimethyl-4H-cyclopenta-1,3-dioxole-4-methanol or 1-l-(1,2,4,5/3)-3-Acetamido-2-amino-1-hydroxymethyl-4,5-O-isopropylidene-4,5-cyclopentanediol **19**

A 5% solution of acetamide **18** (422 mg, 1.27 mmol) in methanol was stirred with Pearlman’s catalyst (Pd(OH)_2_/C, 20%) under an atmosphere of H_2_ at ambient pressure. After completed conversion (1 hour), the catalyst was filtered off, the filtrate was concentrated under reduced pressure, and the residue was chromatographically purified (chloroform/methanol/NH_4_OH (25%) 14:1:0.01 *v*/*v*/*v*) to obtain intermediate **19** as a pale yellow syrup (253 mg, 1.04 mmol, 81.6%). ${\left[\alpha \right]}_{D}^{20}:$ +7.5 (*c* = 0.85, CHCl_3_); ^1^H-NMR (300 MHz, CDCl_3_) δ = 7.29 (d, 1H, NHCOCH_3_), 4.68 (dd, 1H, *J*_3,4_ = 6.0 Hz, *J*_4,5_ = 5.7 Hz, H-4), 4.47 (d, 1H, H-3), 4.05 (d, 1H, *J*_1,2_ = 6.5 Hz, *J*_4,5_ < 1 Hz, H-2), 3.90 (m, 2H, H-6a, H-6b), 3.25 (d, 1H, *J*_1,5_ < 1 Hz, H-1), 3.00 (bs, 3H, 6-OH, 1-NH_2_), 2.41 (m, 1H, H-5), 1.95 (s, 3H, NHCOCH_3_), 1.45, 1.25 (2s, 3H each, C(CH_3_)_2_). ^13^C-NMR (75.5 MHz, CDCl_3_): δ = 170.7 (NHCOCH_3_), 111.0 (C(CH_3_)_2_), 85.4 (C-3), 80.8 (C-4), 63.2 (C-2), 59.3 (C-1), 58.1 (C-6), 47.2 (C-5), 26.5, 23.2 (C(CH_3_)_2_), 23.1 (NHCOCH_3_).

MS (MALDI): Calcd for [C_11_H_20_N_2_O_4_H]: *m/z* 245.1501 [M + H]^+^; Found [M + H]^+^ 245.1506.

### 3.7. (1S,2R,3S,4R,5R)-3-Acetamido-4-amino-5-hydroxymethylcyclopentanetriol or “1-amino-2-acetamido-2-deoxy-β-d-galacto-cyclopentane” **20**

A solution of compound **19** (34.8 mg, 0.142 mmol) in methanol (1 mL) was treated with HCl (12 M 100µL). After completed deprotection, the solvent was removed under reduced pressure, and the remaining residue was purified by silica gel chromatography (chloroform/methanol/NH_4_OH (25%) 8:4:1 *v*/*v*/*v*) to furnish aminopolyol **20** as the free base (22.4 mg, 0.110 mmol, 77.0%). Treatment with HCl_g_ in methanol in the presence of small amounts of ethyl acetate as co-solvent afforded the corresponding hydrochloride (**20**^.^HCl) as a white solid. ${\left[\alpha \right]}_{D}^{20}:$ +57.6 (*c* = 0.90, H_2_O) (hydrochloride); ^1^H-NMR (500 MHz, D_2_O) (free base): δ = 4.21 (dd, 1H, *J*_3,4_ = *J*_4,5_ = 3.7 Hz, H-4), 4.12 (dd, 1H, *J*_1,2_ = 5.7 Hz, *J*_2,3_ = 9.1 Hz, H-2), 4.03 (dd, 1H, *J*_3,4_ = 3.8 Hz, H-3), 3.96 (dd, 1H, *J_5_*_,6_ = 7.4 Hz, *J*_6a,6b_ = 11.2 Hz, H-6a), 3.90 (dd, 1H, *J*_5,6b_ = 7.7 Hz, H-6b), 3.44 (dd, 1H, *J*_1,5_ = 8.7 Hz, H-1), 2.48 (dddd, 1H, *J*_4,5_ = 3.9 Hz, H-5), 2.10 (s, 3H, NHCOCH_3_). ^13^C-NMR (125.9 MHz, D_2_O) (free base): δ = 174.9 (NHCOCH_3_), 75.7 (C-3), 72.2 (C-4), 62.0 (C-2), 57.0 (C-6), 55.6 (C-1), 42.9 (C-5), 21.9 (NHCOCH_3_).

MS (MALDI): Calcd for [C_8_H_16_N_2_O_4_H]: *m/z* 205.1188 [M + H]^+^; Found [M + H]^+^ 2051184.

### 3.8. (1S,2R,3S,4R,5R)-N-(1-Hexyl)-3-acetamido-4-amino-5-hydroxymethylcyclopentanetriol or “2-Acetamido-2-deoxy-1-(hexyl)amino-β-d-galacto-cyclopentane” **21**

Amine **19** (32.2 mg, 0.132 mmol) was dissolved in DMF (1 mL) and treated with 1-bromohexane (22.1 µL, 0.158 mmol) in the presence of NaHCO_3_ (53.2 mg, 0.633 mmol) at 60 °C. After completed consumption of the starting material, the mixture was concentrated under reduced pressure. The residue was diluted with methanol and treated with HCl (100 µL, 12 M) and stirred for one hour. After evaporation of the solvents, the remaining precipiate was purified by chromatography on silica gel (chloroform/methanol/NH_4_OH (25%) 8:1:0.01 *v*/*v*/*v*) to yield *N*-hexyl carbacycle **21** (25.4 mg, 881 µmol, 66.8% over two steps). ${\left[\alpha \right]}_{D}^{20}$: +49.8 (*c* = 0.97, MeOH); ^1^H-NMR (500 MHz, CD_3_OD): δ = 4.16 (dd, 1H, *J*_1,2_ = 5.0 Hz, *J*_2,3_ = 7.7 Hz, H-2), 4.08 (dd, 1H, *J*_3,4_ = *J*_4,5_ = 4.1 Hz, H-4), 3.96 (dd, 1H, *J*_5,6b_ = 7.4 Hz, *J*_6a,6b_ = 11.2 Hz, H-6a), 3.91 (m, 2H, H-3, H-6b), 3.21 (dd, 1H, *J*_1,5_ = 8.2 Hz, H-1), 2.94 (m, 1H, H-1′a), 2.73 (m, 1H, H-1′b), 2.44 (dddd, 1H, H-5), 2.04 (s, 3H, NHCOCH_3_), 1.58 (m, 2H, H-2′), 1.47–1.33 (m, 6H, H-3′, H-4′, H-5′), 0.97 (t, 3H, H-6′). ^13^C-NMR (125.9 MHz, CD_3_OD): δ = 173.3 (NHCOCH_3_), 78.9 (C-3), 74.0 (C-4), 64.6 (C-1), 63.0 (C-2), 59.1 (C-6), 47.9 (C-1′), 45.6 (C-5), 32.7, 29.6, 27.8, 23.6 (C-2′, C-3′, C-4′, C-5′) 22.8 (NHCOCH_3_), 14.3 (C-6′).

MS (MALDI): Calcd for [C_14_H_28_N_2_O_4_H]: *m/z* 289.2127 [M + H]^+^; Found [M + H]^+^ 289.2126.

### 3.9. (1S,2R,3S,4R,5R)-N-(Methoxycarbonyl)pentyl-3-acetamido-4-amino-5-hydroxymethyl-cyclopentanetriol or “2-Acetamido-2-deoxy-1-(methoxycarbonylhexyl)amino-β-d-galacto-cyclopentane” **22**

Amine **19** (25.7 mg, 0.105 mmol) was dissolved in DMF (1 mL) and NaHCO_3_ (42.4 mg, 0.505 mmol) followed by methyl 6-iodohexanoate (20.8 mg, 0.505 mmol) were added. The reaction mixture was heated to 60 °C until completed consumption of the starting material was observed (tlc). The mixture was then concentrated under reduced pressure, and MeOH was added to obtain a ca. 50% solution. This was added to a mixture of methanol (5 mL) and acetic chloride (100 µL) at 0 °C, and the mixture was stirred for 15 min. Removal of solvent *in vacuo* and followed by chromatography of the residue (chloroform/methanol/NH_4_OH (25%) 8:1:0.01 *v*/*v*/*v*) gave free methyl ester **22** (23.8 mg, 71.6 µmol, 68.1% over two steps) as a colourless syrup. ${\left[\alpha \right]}_{D}^{20}$: +41.0 (*c* = 0.99, MeOH); ^1^H-NMR (300 MHz, CD_3_OD): δ = 4.07 (dd, 1H, *J*_1,2_ = 5.0 Hz, *J*_2,3_ = 7.4 Hz, H-2), 4.02 (dd, 1H, *J*_3,4_ = *J*_4,5_ = 4.1 Hz, H-4), 3.97–3.80 (m, 3H, H-3, H-6), 3.66 (s, 3H, H-1″), 3.06 (dd, 1H, *J*_1,5_ = 8.1 Hz, H-1), 2.83 (m, 1H, H-1′a), 2.56 (m, 1H, H-1′b), 2.42 – 2.28 (m, 3H, H-5, H-5′), 1.98 (s, 3H, NHCOCH_3_), 1.72–1.28 (m, 6H, H-2′, H-3′, H-4′). ^13^C-NMR (75.5 MHz, CD_3_OD): δ = 175.8 (COOMe), 173.1 (NHCOCH_3_), 79.1 (C-3), 74.0 (C-4), 64.5 (C-1), 63.6 (C-2), 59.1 (C-6), 52.0 (C-1″), 47.9 (C-1′), 46.0 (C-5), 34.7, 29.9, 27.7, 25.8 (C-2′, C-3′, C-4′, C-5′) 22.8 (NHCOCH_3_).

MS (MALDI): Calcd for [C_15_H_28_N_2_O_6_H]: *m/z* 333.2026 [M + H]^+^; Found [M + H]^+^ 333.2024.

### 3.10. (1S,2R,3S,4R,5R)-N-(6-Amino)hexyl-3-acetamido-4-amino-5-hydroxymethyl-cyclopentanetriol or “2-Acetamido-2-deoxy-1-(6-aminohexyl)amino-β-d-galacto-cyclopentane” **24**

Amine **19** (68.2 mg, 0.279 mmol) was dissolved in DMF (3 mL) and treated with 6-bromohexanoic nitrile (44.4 µL, 0.335 mmol) in the presence of NaHCO_3_ (93.8 mg, 1.34 mmol). The reaction mixture was heated to 60 °C until completed consumption of the starting material was observed. The solvents were removed under reduced pressure. The residue was diluted with methanol, 2 M HCl (100 µL) was added, and the mixture was stirred for one hour. After evaporation of the solvents, the residue was purified by column chromatography (chloroform/methanol/NH_4_OH (25%) 8:1:0.01 *v*/*v*/*v*) to yield nitrile **23** (30.9 mg, 0.102 mmol, 37.0% from compound **19**), which was directly used in the next step. ${\left[\alpha \right]}_{D}^{20}$: +24.3 (*c* = 0.975, MeOH); ^1^H-NMR (300 MHz, CD_3_OD): δ = 4.32 (dd, 1H, *J*_1,2_ = 5.2 Hz, *J*_2,3_ = 7.2 Hz, H-2), 4.10–3.94 (m, 4H, H-3, H-4, H-6a, H-6b), 3.64 (dd, 1H, *J*_1,5_ = 7.8 Hz, H-1), 3.23 (m, 2H, H-1′), 2.61 (m, 1H, H-5), 2.51 (t, 2H, H-5′), 2.05 (s, 3H, NHCOCH_3_), 1.84–1.45 (m, 6H, H-2′, H-3′, H-4′). ^13^C-NMR (75.5 MHz, CDCl_3_): δ = 174.1 (NHCOCH_3_), 121.0 (CN), 77.8 (C-3), 73.8 (C-4), 64.5 (C-1), 60.3 (C-2), 58.5 (C-6), 48.0 (C-1′), 44.5 (C-5), 26.6, 26.0, 25.9, (C-2′, C-3′, C-4′) 22.8 (NHCOCH_3_), 17.2 (C-5′).

A 10% solution of nitrile **23** (30.9 mg, 0.102 mmol) in methanol was stirred with small amounts of Raney-Ni under an atmosphere of H_2_ at ambient temperature. After full conversion of the starting material (20 min), the catalyst was filtered off, and the filtrate was concentrated under reduced pressure. Chromatographic purification (chloroform/methanol/NH_4_OH (25%) 8:4:1 *v*/*v*/*v*) afforded amine **24** as pale yellow syrup (30.9 mg, 0.102 mmol, 77.4%). Treatment with HCl_g_ provided the corresponding dihydrochloride **24·**HCl as a white solid. ${\left[\alpha \right]}_{D}^{20}$: +38.5 (*c* = 1.115, H_2_O) (hydrochloride); ^1^H-NMR (300 MHz, CD_3_OD) (free base): δ = 4.14–4.03 (m, 2H, H-2, H-4), 3.86 (dd, 1H, *J*_2,3_ = 8.5 Hz, *J*_3,4_ = 4.2 Hz, H-3), 3.81 (d, 2H, H-6), 3.08 (dd, 1H, *J*_1,2_ = 5.6 Hz, *J*_1,5_ = 8.9 Hz, H-1), 2.76–2.56 (m, 3H, H-6′a, H-6′b, H-1′a), 2.47–2.34 (m, 2H, H-5, H-1′b), 2.00 (s, 3H, NHCOCH_3_), 1.57–1.21 (m, 8H, H-2′, H-3′, H-4′, H-5′). ^13^C-NMR (75.5 MHz, D_2_O) (free base): δ = 173.2 (NHCOCH_3_), 77.1 (C-3), 72.0 (C-4), 61.5 (C-2), 60.8 (C-1), 57.4 (C-6), 46.5 (C-1′), 43.8 (C-5), 40.2, 30.3, 28.0, 26.2, 25.7 (C-2′, C-3′, C-4′, C-5′, C-6′) 22.2 (NHCOCH_3_).

MS (MALDI): Calcd for [C_14_H_29_N_3_O_4_H]: *m/z* 304.2236 [M + H]^+^; Found [M + H]^+^ 304.2234.

### 3.11. (1S,2R,3S,4R,5R)-N-(6-Dansylamino)hexyl-3-acetamido-4-amino-5-hydroxymethyl-cyclopentanetriol or “2-Acetamido-2-deoxy-1-(6-dansylaminohexyl)amino-β-d-galacto-cyclopentane” **25**

A solution of amine **24** (23.2 mg, 61.6 µmol) in methanol (1mL) was treated with Et_3_N (38.5 µL, 277 mmol) and dansyl chloride (18.3 mg, 67.8 µmol). After completed conversion of the starting material (30 min), the solvent was removed under reduced pressure. Purification on silica gel (chloroform/methanol/NH_4_OH (25%) 8:1:0.01 *v*/*v*/*v*) provided compound **25** (16.1 mg, 48.6 µmol, 78.9%) as light yellow, fluorescent syrup. ${\left[\alpha \right]}_{D}^{20}$: +23.7 (*c* = 0.960, MeOH); ^1^H-NMR (300 MHz, CD_3_OD): δ = 4.07 (dd, 1H, *J*_1,2_ = 4.9 Hz, *J*_2,3_ = 7.6 Hz, H-2), 4.01 (dd, 1H, *J*_3,4_ = *J*_4,5_ = 4.0 Hz, H-4), 3.93–3.79 (m, 3H, H-3, H-6a, H-6b), 3.09 (dd, 1H, *J*_1,5_ = 8.1 Hz, H-1), 2.90–2.71 (m, 3H, H-1’a, H-6’a, H-6’b), 2.53 (m, 1H, H-1’b), 2.35 (ddd, 1H, *J*_5,6a_ = *J*_5,6b_ = 7.2 Hz), 1.97 (s, 3H, NHCOCH_3_), 1.40–1.05 (m, 8H, H-2′, H-3′, H-4′, H-5′). ^13^C-NMR (75.5 MHz, CD_3_OD): δ = 173.2 (NHCOCH_3_), 78.9 (C-3), 74.0 (C-4), 64.6 (C-1), 63.1 (C-2), 59.0 (C-6), 47.9 (C-1′), 45.6 (C-5), 43.7, 30.4, 29.5, 27.5, 27.2 (C-2′, C-3′, C-4′, C-5′, C-6′) 22.8 (NHCOCH_3_).

MS (MALDI): Calcd for [C_26_H_40_N_4_O_6_SH]: *m/z* 537.2747 [M+H]^+^; Found [M+H]^+^ 537.2750.

## Figures and Tables

**Table 1:** Inhibitory activities of new compounds with *N*-acetylhexosaminidases.

**Enzyme**	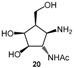	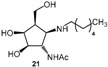	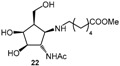	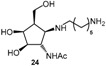	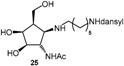
SpHex	0.0007	0.0006	0.0007	0.0007	0.00006
HexA (h.lys.)	n.d.	0.88	n.d.	n.d.	0.30

^1^*K*_i_-values [μM] of compounds with: SpHex = *Streptomyces plicatus N*-acetyl-β-d-hexosaminidase; HexA h. lys. = IC_50_ [μM] with human lysosomal *N*-acetyl-β-d-hexosaminidase A.; n.d., not determined.

## Supplementary Materials

NMR spectra of compounds are available online.
