# Supplementary material for: Potent GH20 N-Acetyl-β-d-hexosaminidase Inhibitors: N-Substituted 3-acetamido-4-amino-5-hydroxymethyl-cyclopentanediols
Source: Molecules. 2018 Mar 20;23(3):708. doi: 10.3390/molecules23030708 (PMC6017319; doi:10.3390/molecules23030708)

Potent GH20 *N*-Acetyl-b-D-hexosaminidase inhibitors: N-Substituted 4-acetamido-5-amino- 1-hydroxymethyl-cyclopentanediols

Patrick Weber[1], Seyed A. Nasseri[2], Bettina M. Pabst[3], Ana Torvisco[4], Philipp Müller[4], Eduard Paschke[3], Marion Tschernutter[3], Werner Windischhofer[3], Stephen G. Withers[2], Tanja M. Wrodnigg[1], Arnold E. Stütz[1*]

[1] Glycogroup, Institute of Organic Chemistry, Graz University of Technology, Stremayrgasse 9, A-8010 Graz, Austria; patrick.weber@tugraz.at (P.W.); t.wrodnigg@tugraz.at (T.M.W.)

[2] Chemistry Department, University of British Columbia, 2036 Main Mall, Vancouver, BC V6T 1Z1, Canada; snasseri@chem.ubc.ca (S.A.N.); withers@chem.ubc.ca (S.G.W.)

[3] Laboratory of Metabolic Diseases, Department of Pediatrics, MedUni Graz, Auenbruggerplatz 30, A-8036 Graz, Austria; bettina.pabst@medunigraz.at (B.M.P.); eduard.paschke@inode.at (E.P.); marion.tschernutter@medunigraz.at (M.T.); werner.windischhofer@medunigraz.at (W.W.)

[4] Institute of Inorganic Chemistry, Graz University of Technology, Stremayrgasse 9, A-8010 Graz, Austria; ana.torviscogomez@tugraz.at (A.T.); philipp.mueller@tugraz.at (P.M.)

(3a*R*,3b*S*,6a*R*,7*R*,7a*R*)-Hexahydro-5,5-dimethyl-1-phenyl-1H-[1,3]dioxolo[3,4]cyclopent [1,2-c]isoxazol-7-ol or **1-L-(1,2,3,4,5)-1^1,2^1-anhydro-1-hydroxymethyl-2-(*N*-hydroxy) benzylamino-4,5-*O*-isopropylidene-3,4,5-cyclopentanetriol 14**

^{1}H NMR (300 MHz, $CDCl_3$): compound **14**

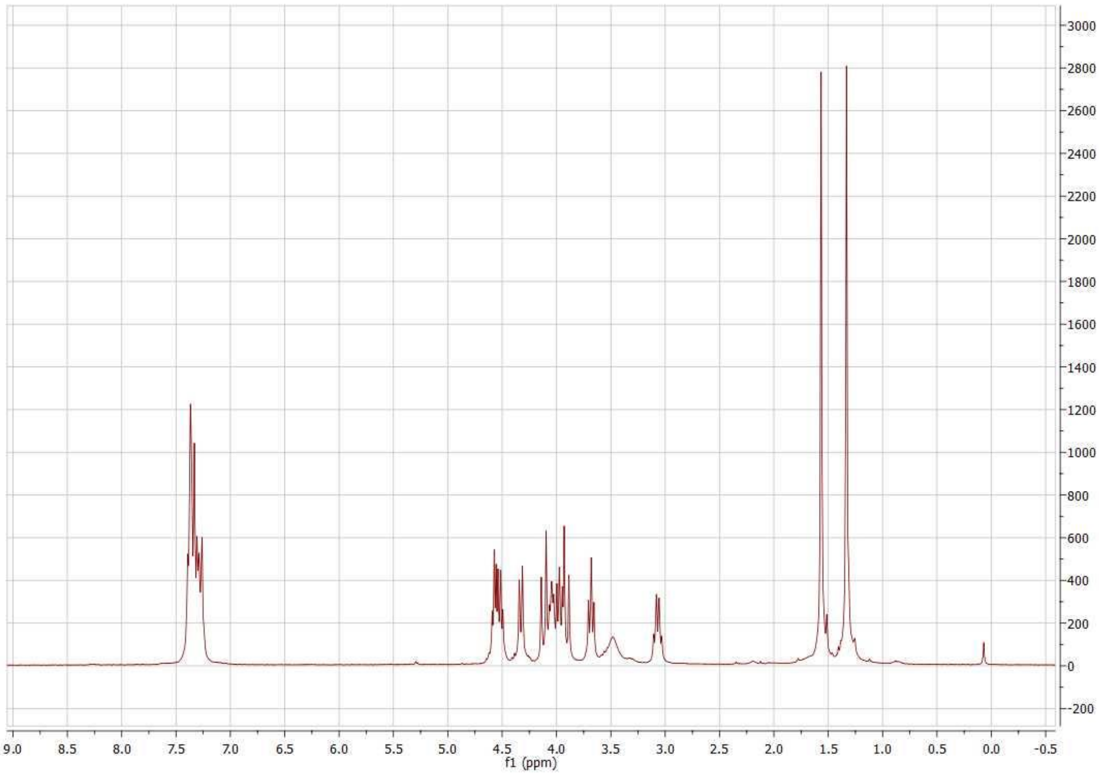

^{13}C NMR (75.5 MHz, $CDCl_3$): compound **14**

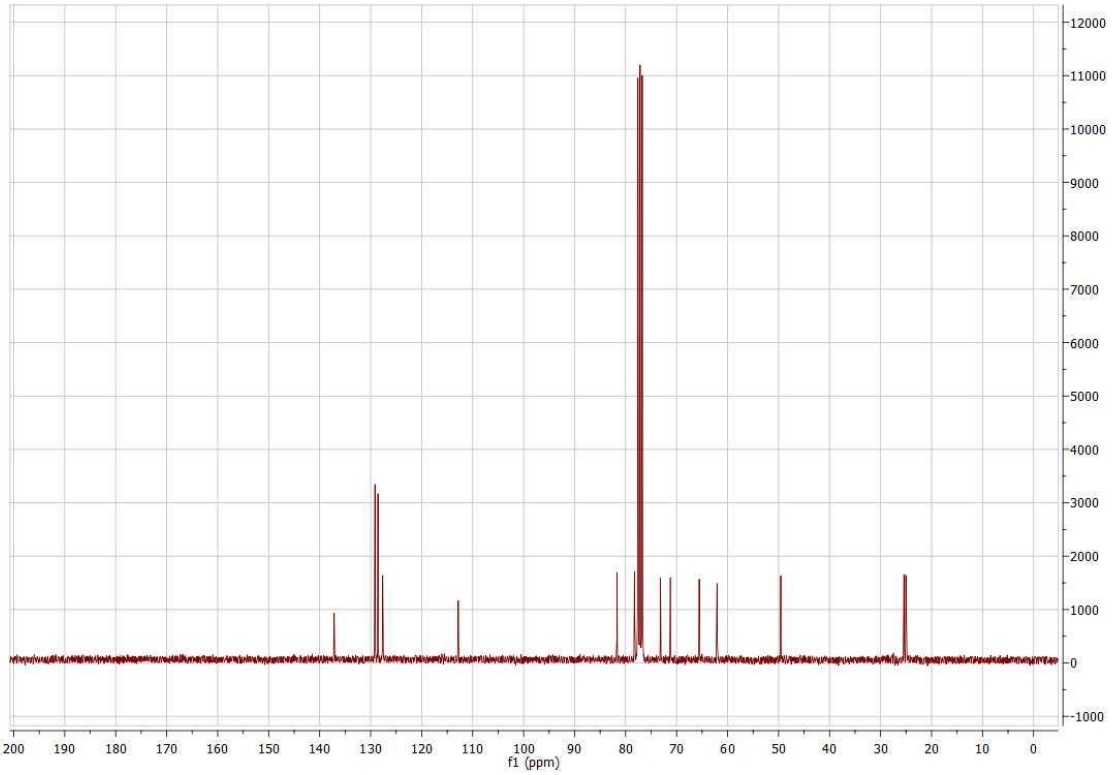

COSY ($CDCl_3$): compound **14**

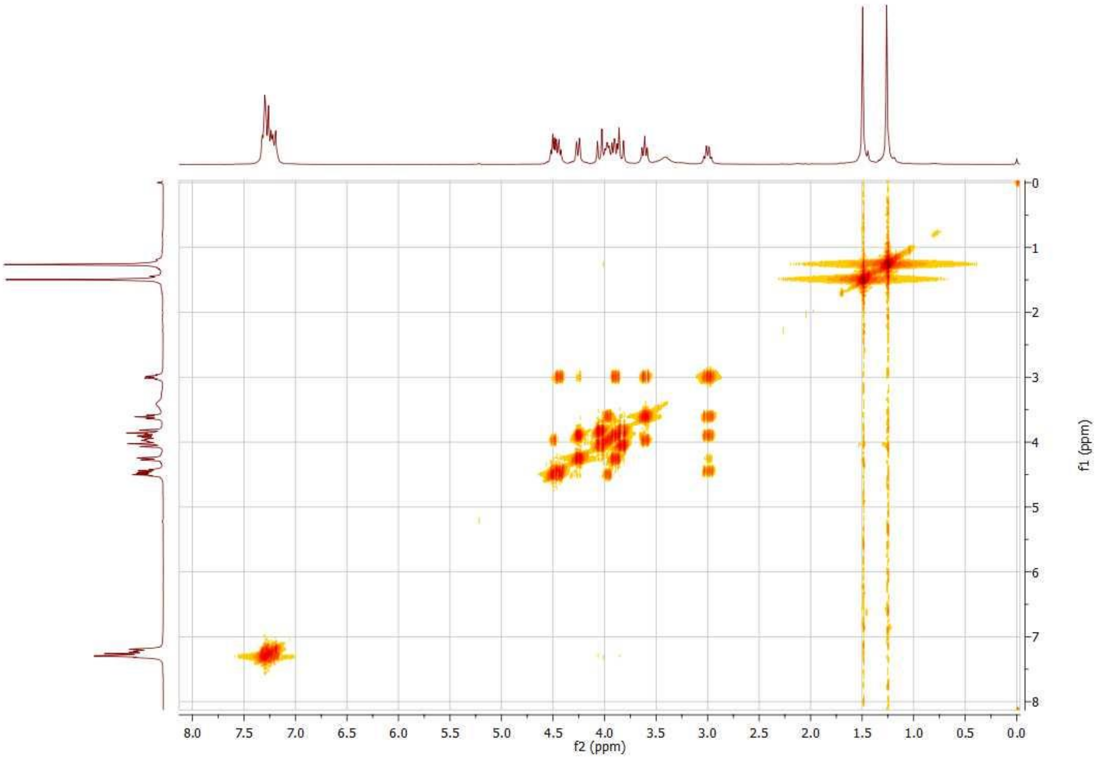

HSQC ($CDCl_3$): compound **14**

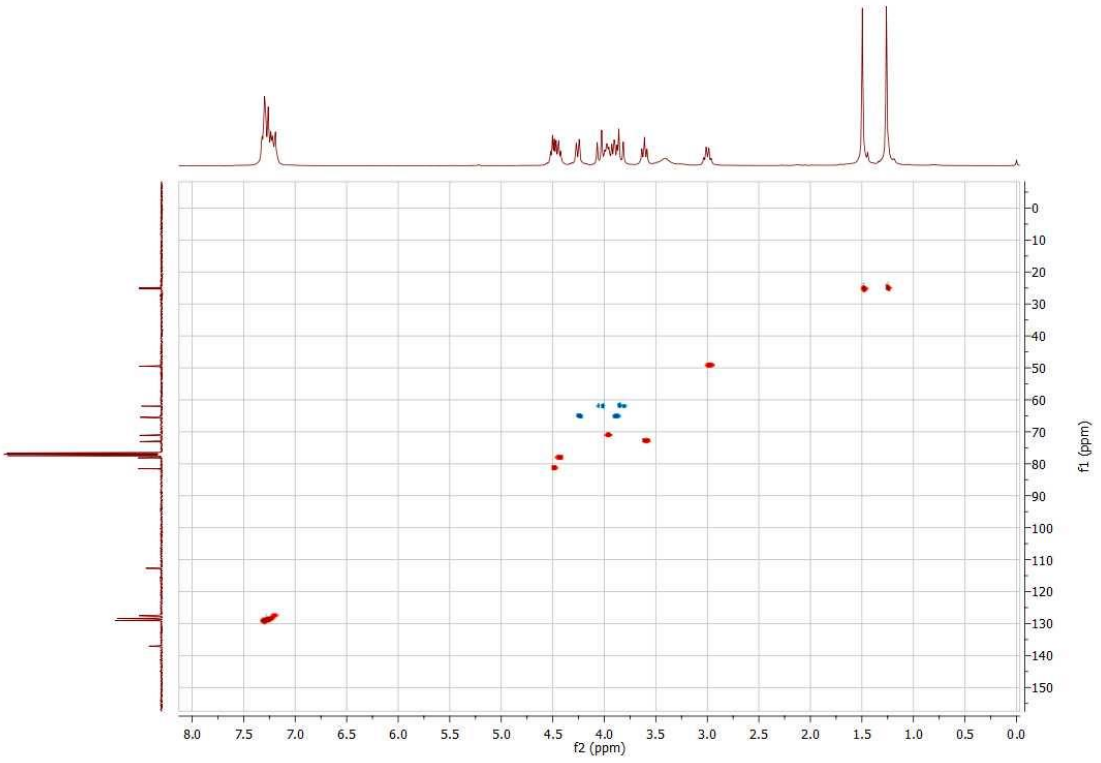

(3a*R*,3b*S*,6a*R*,7*S*,7a*R*)-Hexahydro-7-azido-5,5-dimethyl-1-phenyl-1H-[1,3]dioxolo [3,4]cyclopent[1,2-c]isoxazol or **1-L-(1,2,4,5/3)-1^1,2^1-anhydro-3-azido-1-hydroxymethyl-2-(*N*-hydroxy)benzylamino-4,5-*O*-isopropylidene-4,5-cyclopentanediol 16**

^{1}H NMR (300 MHz, $CDCl_3$): compound **16**

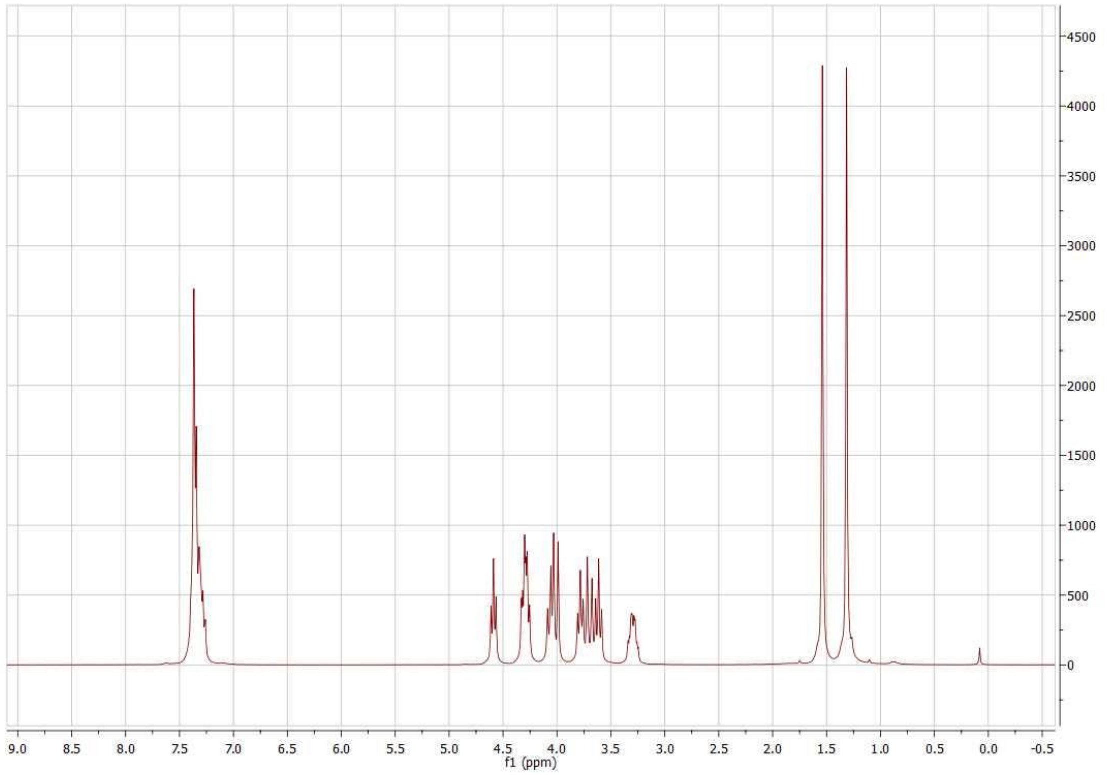

^{13}C NMR (75.5 MHz, $CDCl_3$): compound **16**

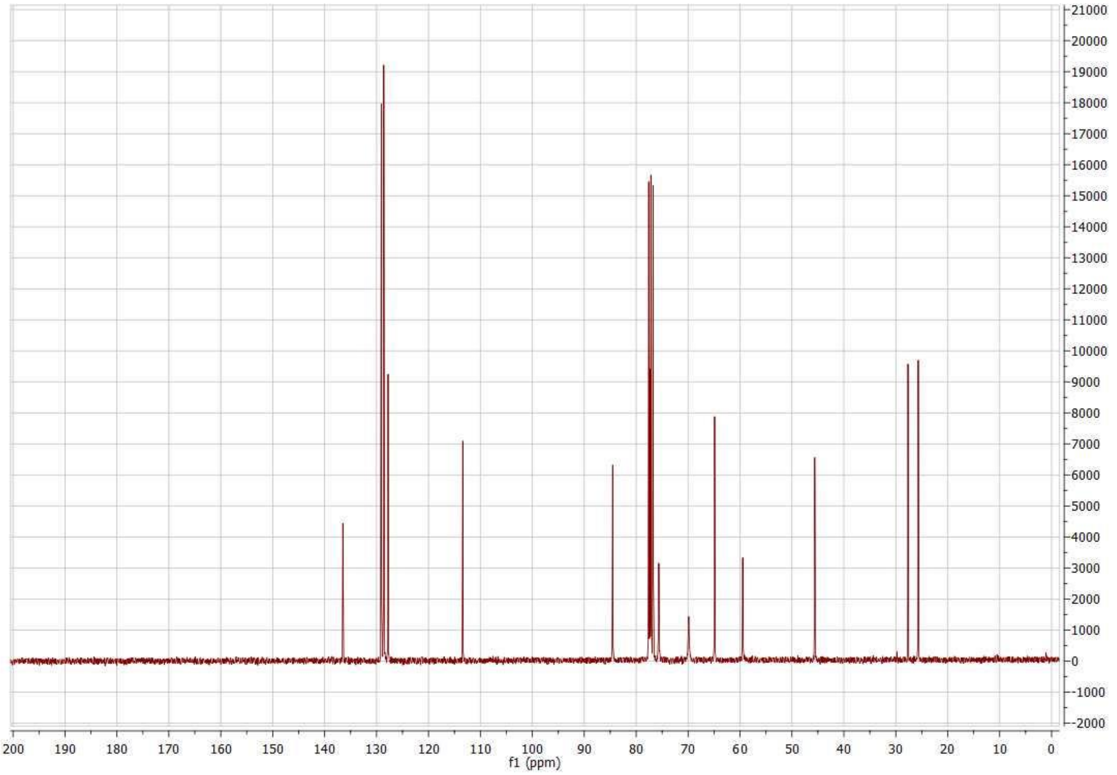

COSY ($CDCl_3$): compound **16**

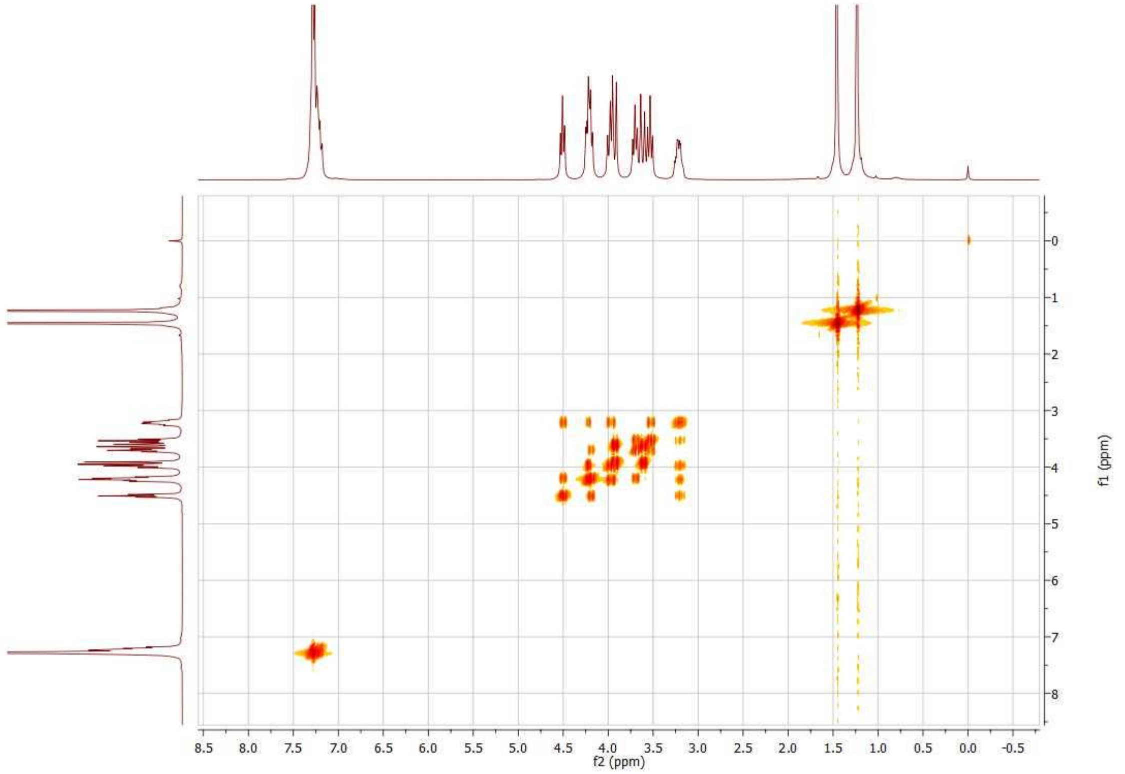

HSQC ($CDCl_3$): compound **16**

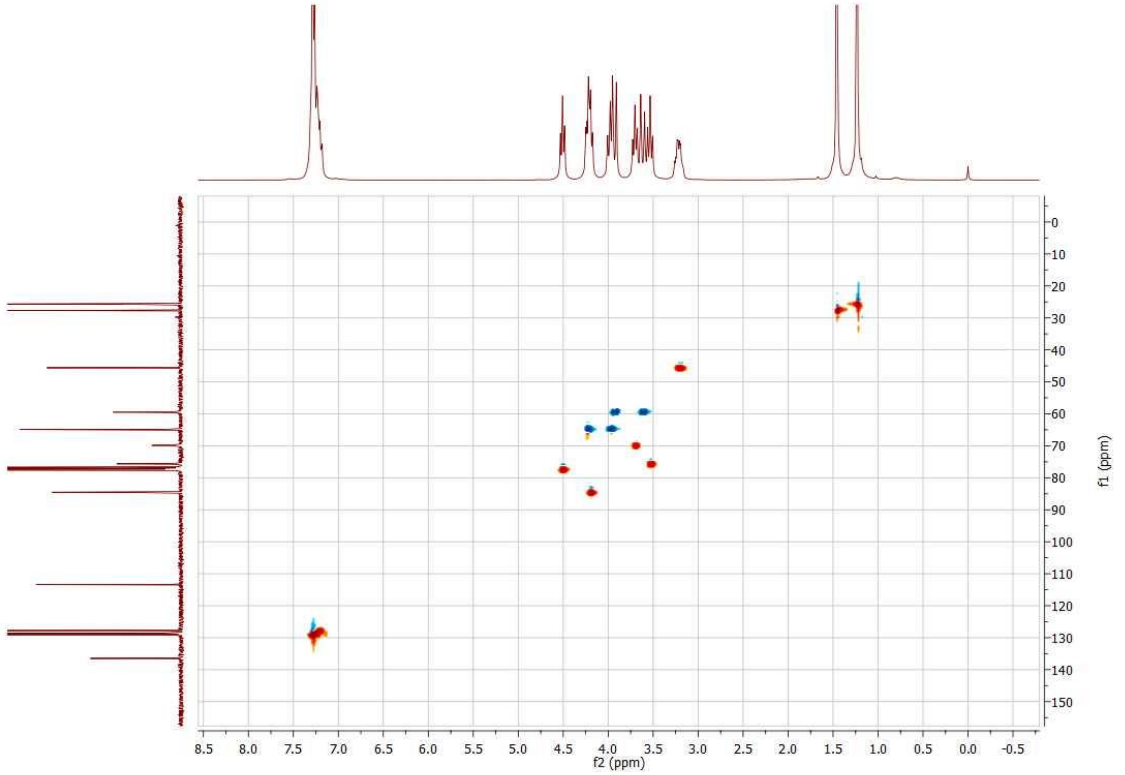

(3a*R*,3b*S*,6a*R*,7*S*,7a*R*)-Hexahydro-7-acetamido-5,5-dimethyl-1-phenyl-1H-[1,3]dioxolo [3,4]cyclopent[1,2-c]isoxazol or **1-L-(1,2,4,5/3)-1^{1},2^{1}-anhydro-3-acetamido-1-hydroxymethyl-2-(*N*-hydroxy)benzylamino-4,5-*O*-isopropylidene-4,5-cyclopentanediol 18**

^{1}H NMR (300 MHz, $CDCl_3$): compound **18**

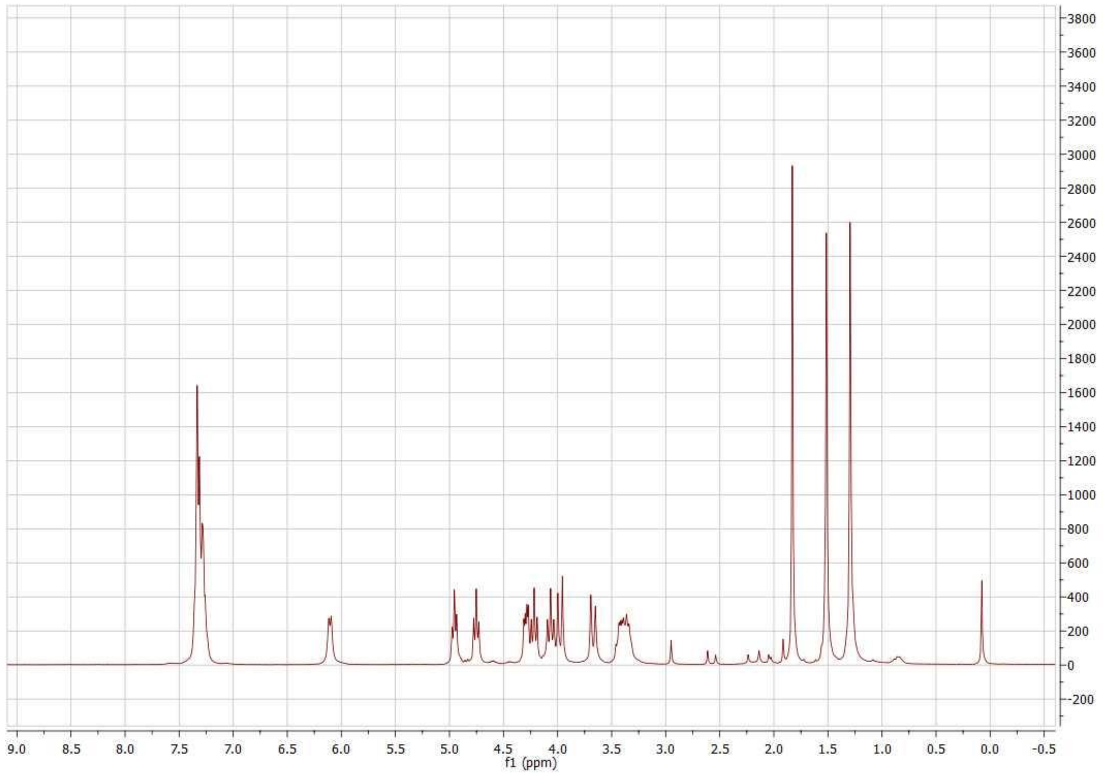

^{13}C NMR (75.5 MHz, $CDCl_3$): compound **18**

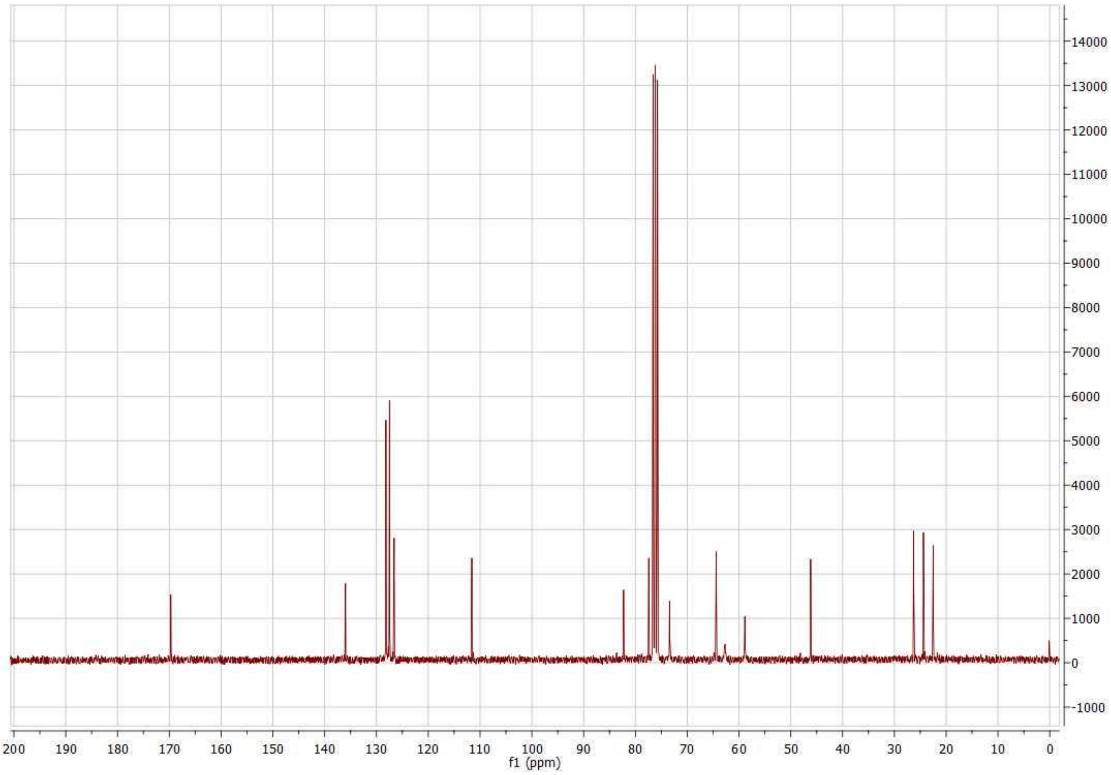

COSY ($CDCl_3$): compound **18**

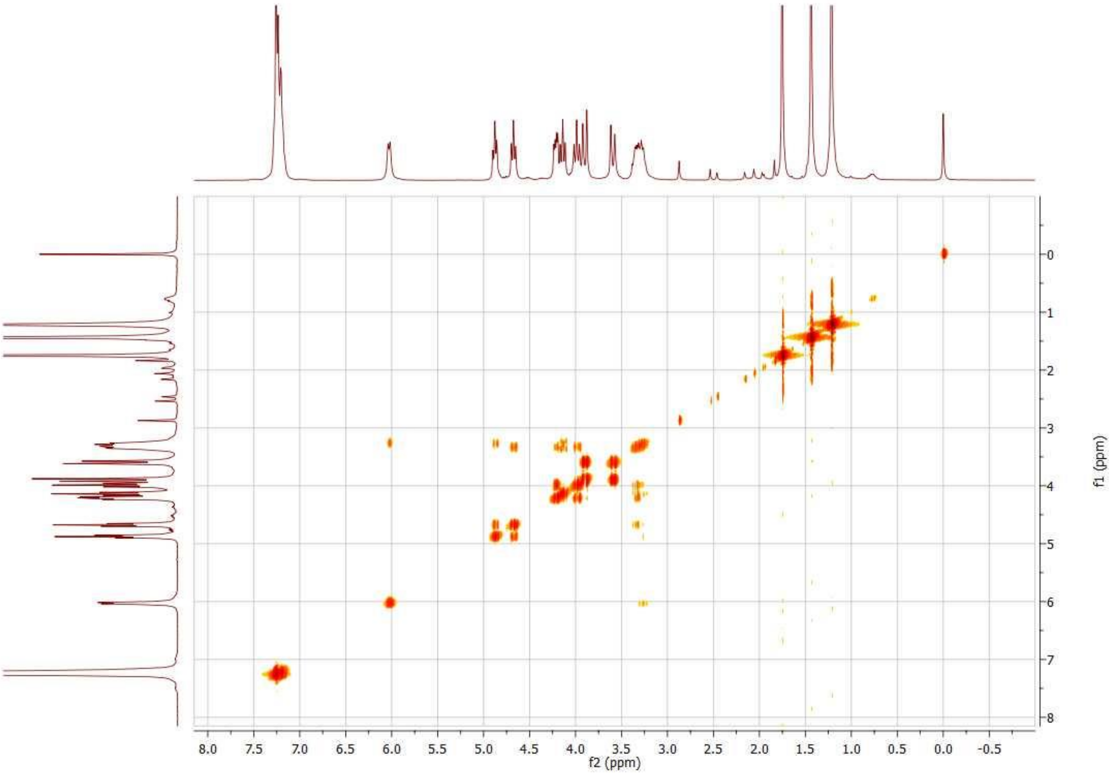

HSQC ($CDCl_3$): compound **18**

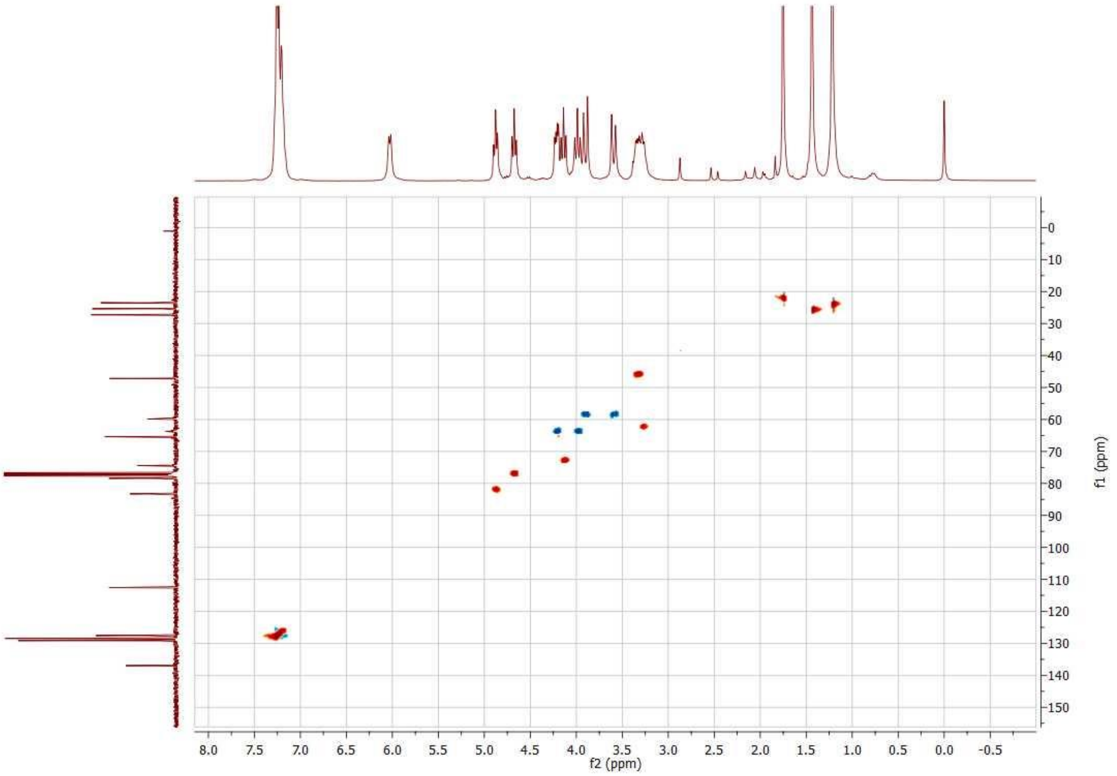

(3a*S*,4*R*,5*R*,6*S*,6a*R*)-5-Amino-tetrahydro-6-acetamido-2,2-dimethyl-4H-cyclopenta-1,3-dioxole-4-methanol or 1-L-(1,2,4,5/3)-3-acetamido-2-amino-1-hydroxymethyl-4,5-*O*-isopropylidene-4,5-cyclopentanediol 19

^{1}H NMR (300 MHz, $CDCl_3$): compound **19**

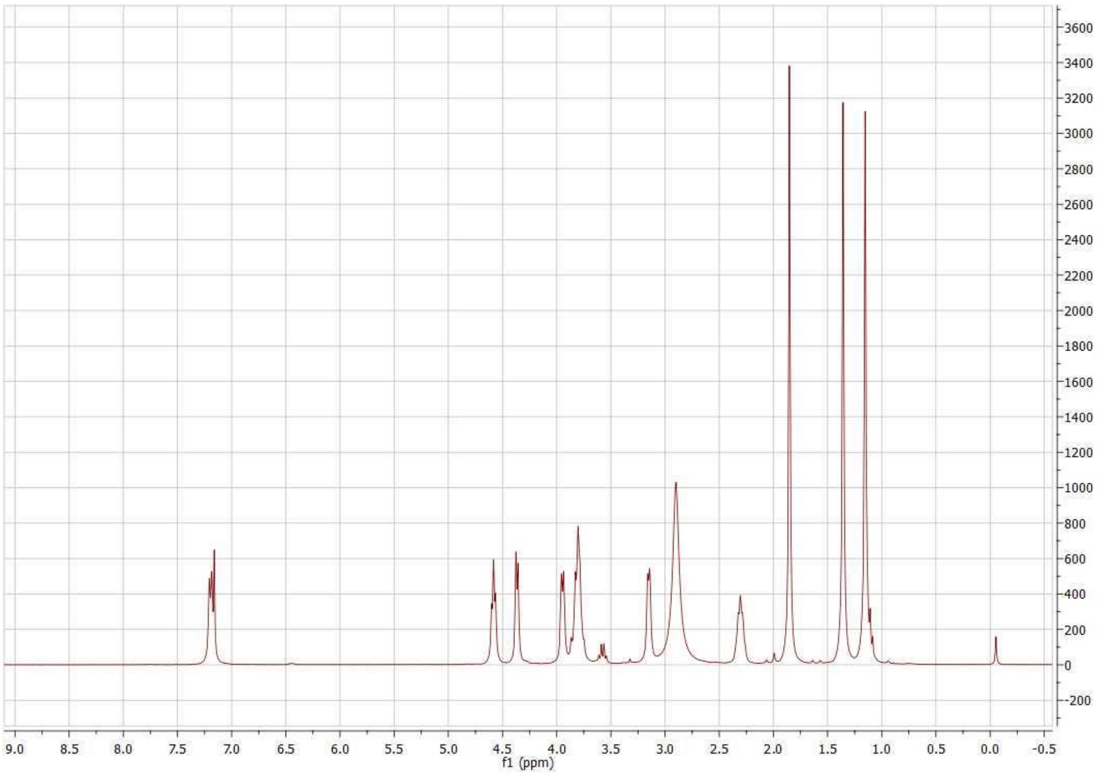

^{13}C NMR (75.5 MHz, $CDCl_3$): compound **19**

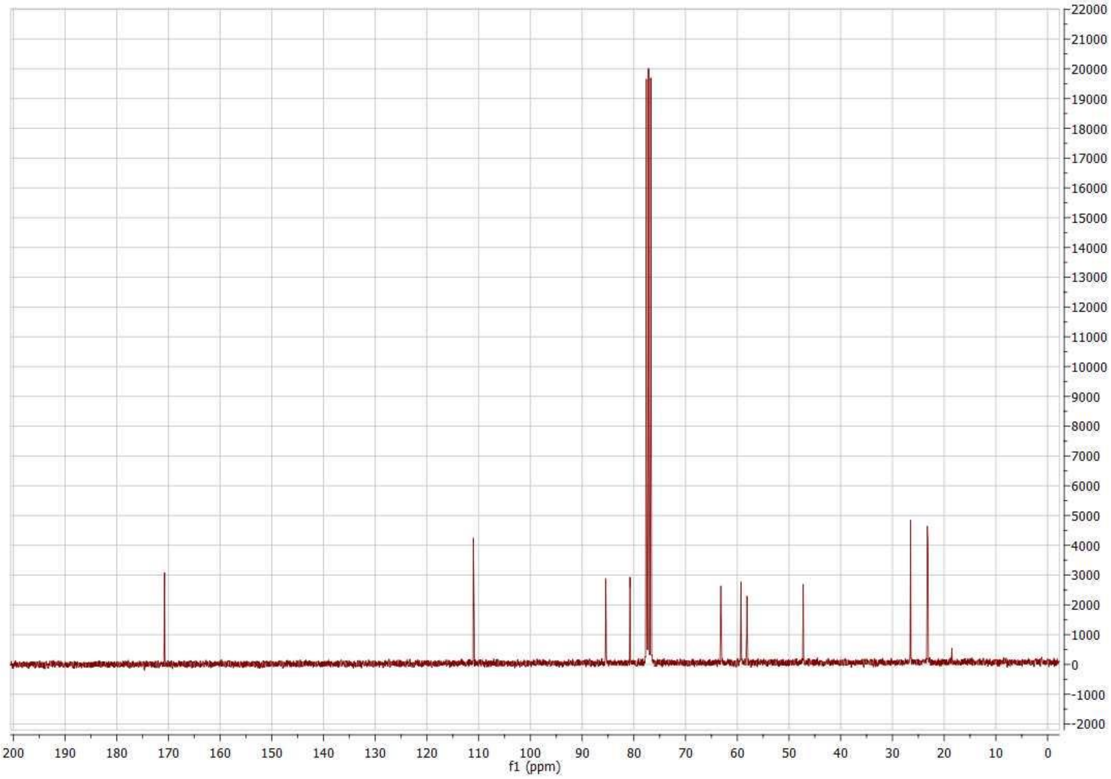

COSY ($CDCl_3$): compound **19**

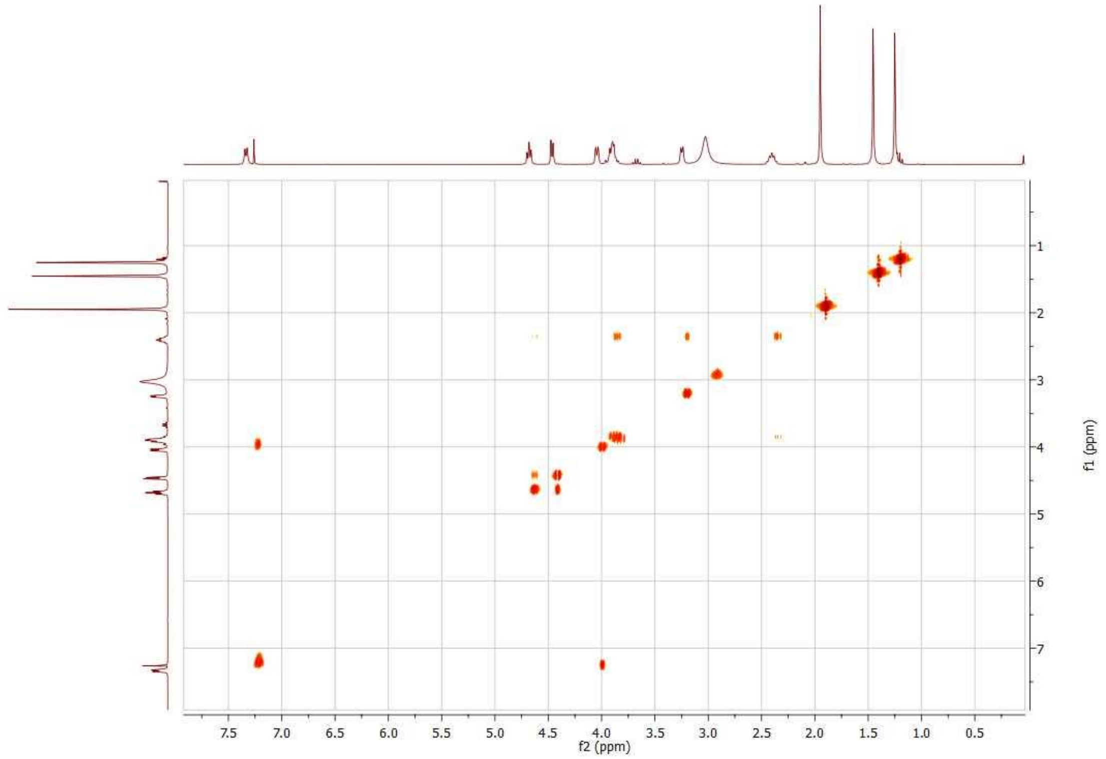

HSQC ($CDCl_3$): compound **19**

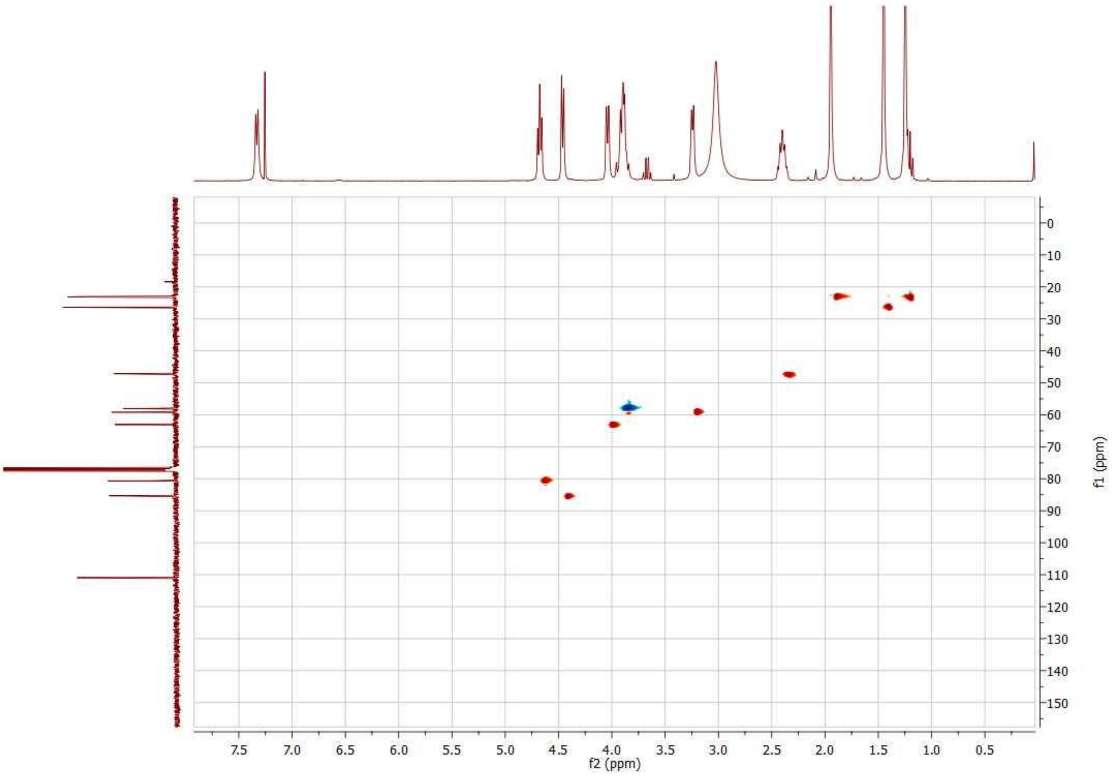

(1*S*,2*R*,3*S*,4*R*,5*R*)-3-Acetamido-4-amino-5-hydroxymethylcyclopentanetriol or "**1-amino-2-acetamido-2-deoxy-β-D-*galacto*-cyclopentane**" **20**

^{1}H NMR (500 MHz, D_2O): compound **20**

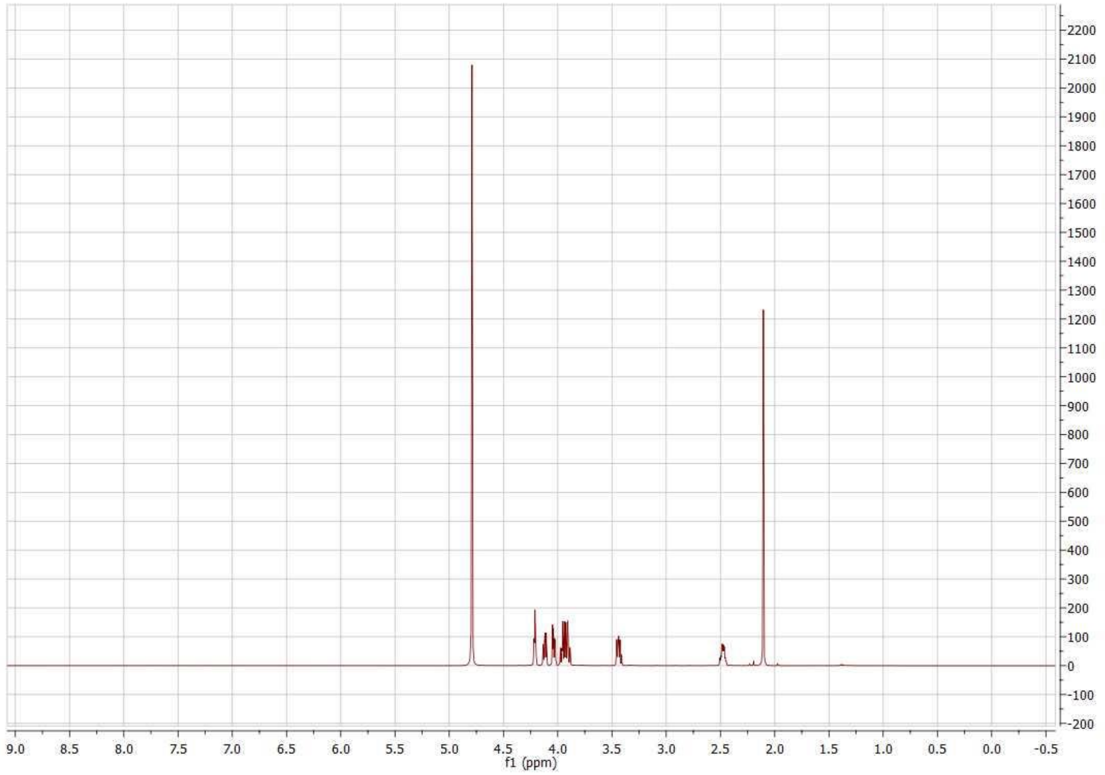

^{13}C NMR (125.9 MHz, D_2O): compound **20**

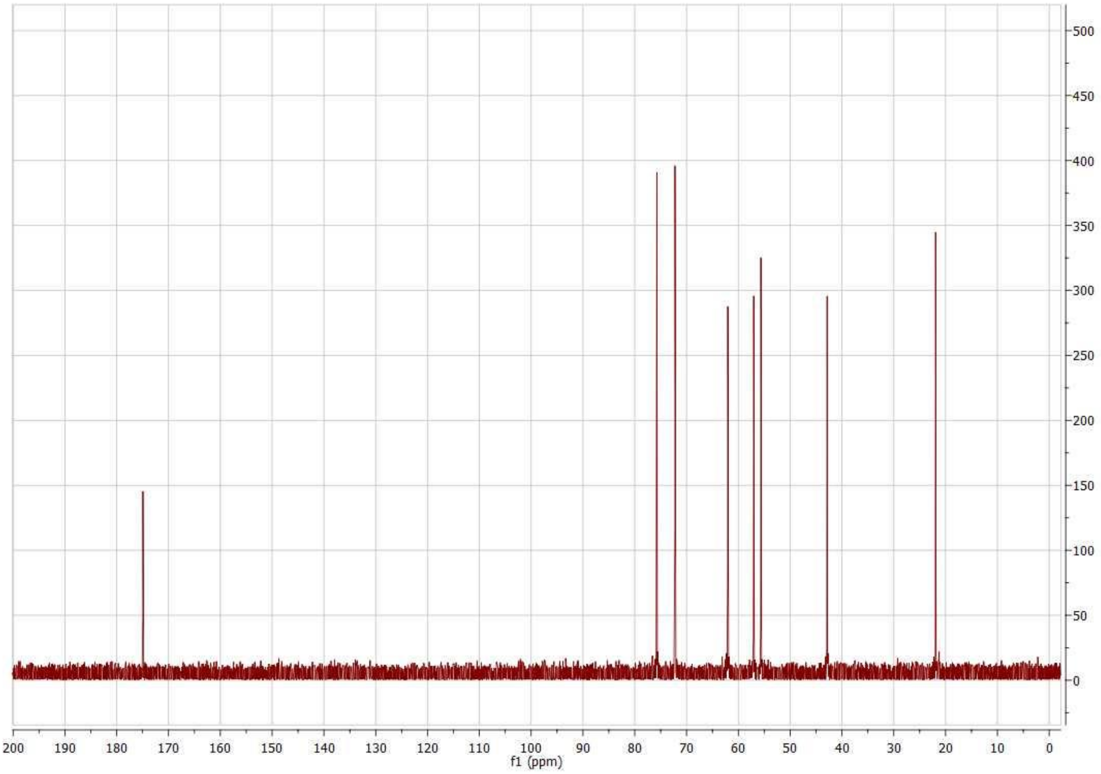

COSY (D_2O): compound **20**

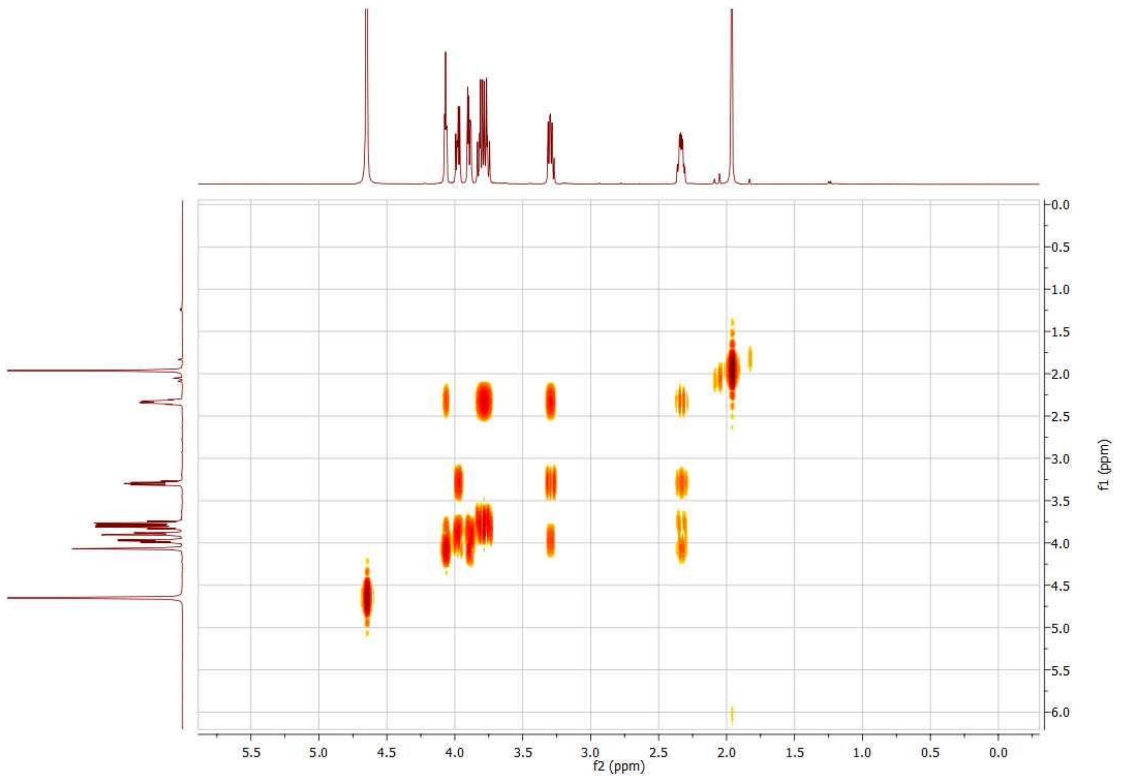

HSQC (D_2O): compound **20**

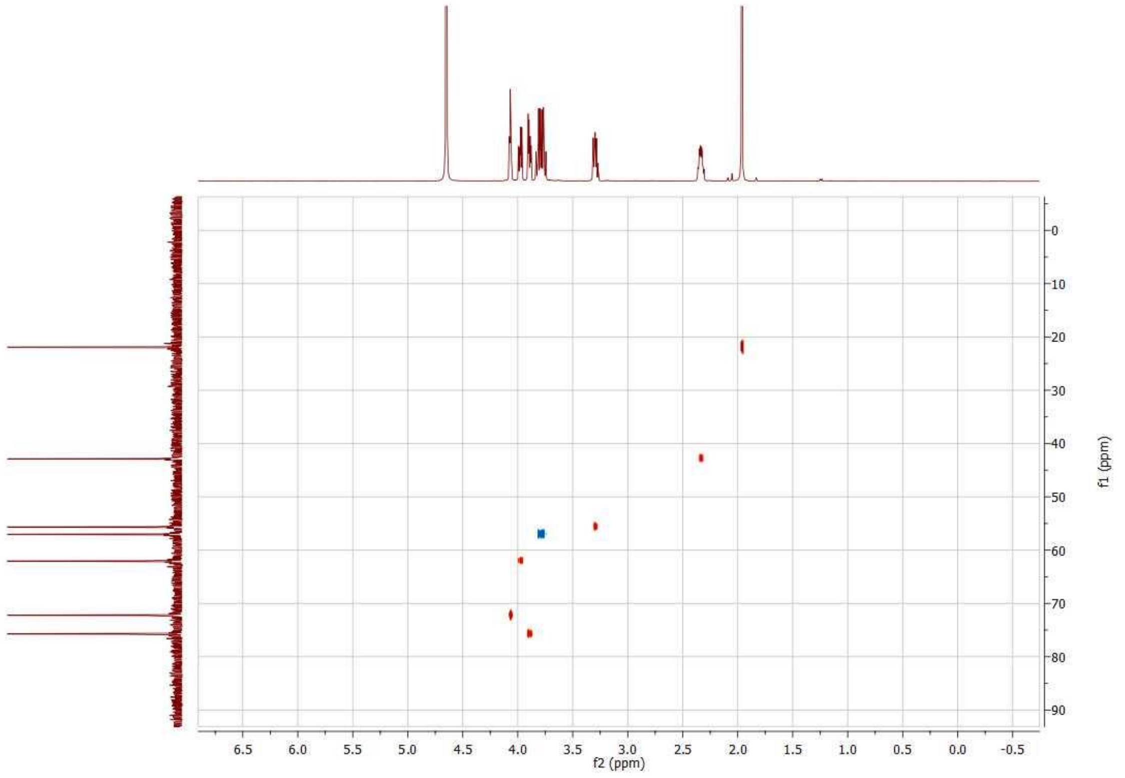

(1*S*,2*R*,3*S*,4*R*,5*R*)-*N*-(1-Hexyl)-3-acetamido-4-amino-5-hydroxymethylcyclopentanetriol or "**2-acetamido-2-deoxy-1-(hexyl)amino-β-D-*galacto*-cyclopentane**" **21**

^{1}H NMR (500 MHz, CD_3OD): compound **21**

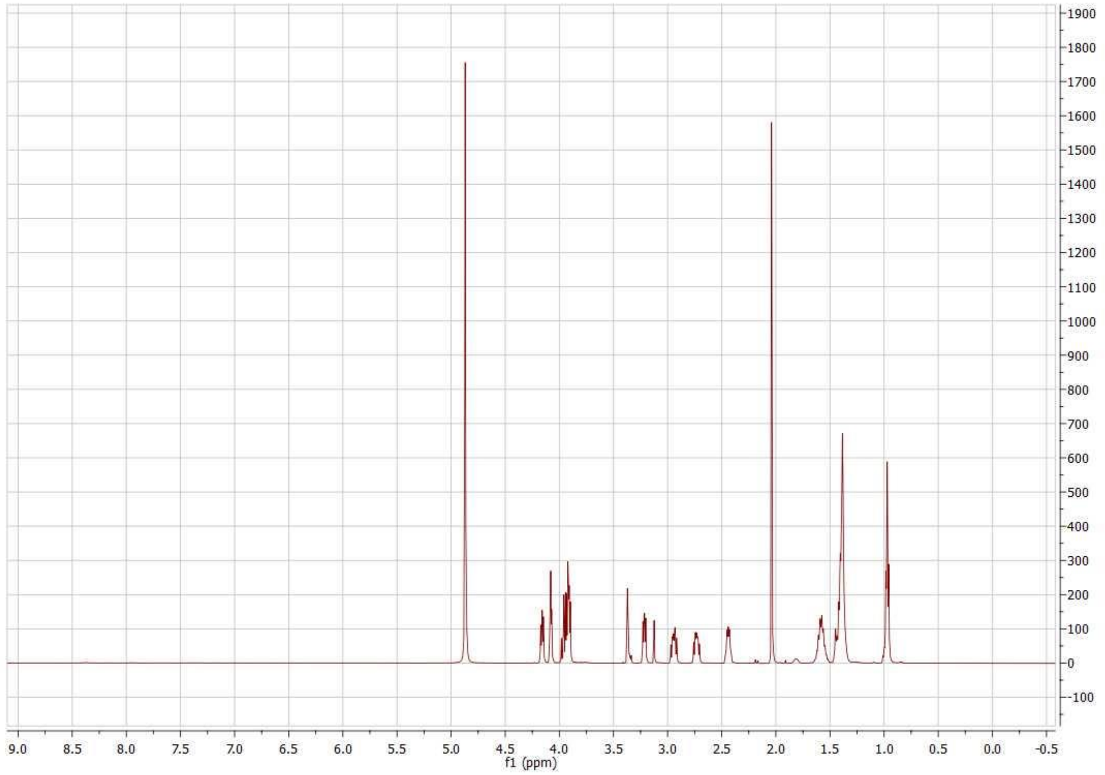

^{13}C NMR (125.9 MHz, CD_3OD): compound **21**

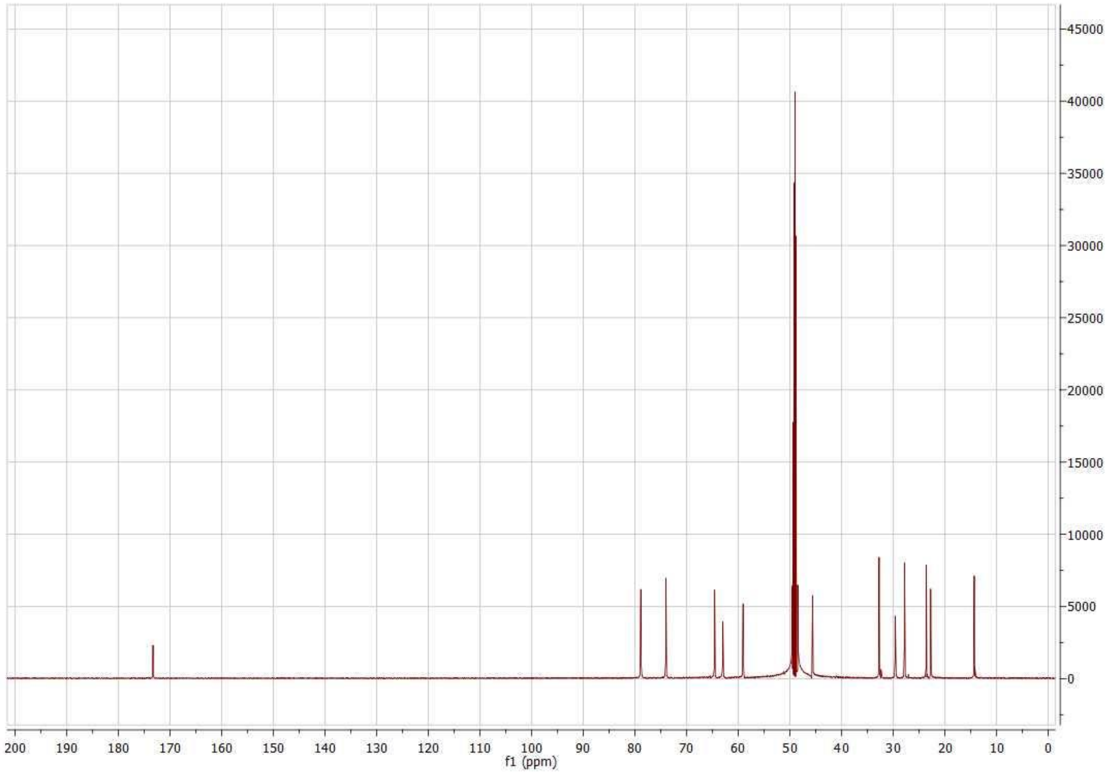

COSY (CD_3OD): compound **21**

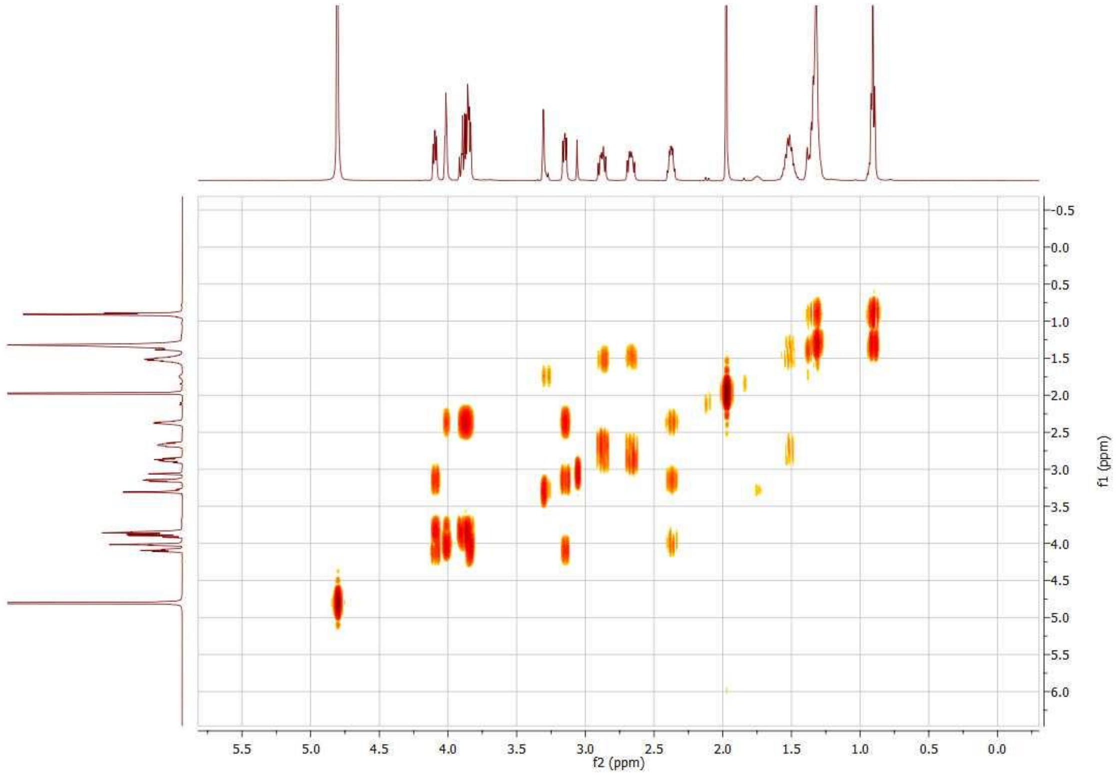

HSQC (CD_3OD): compound **21**

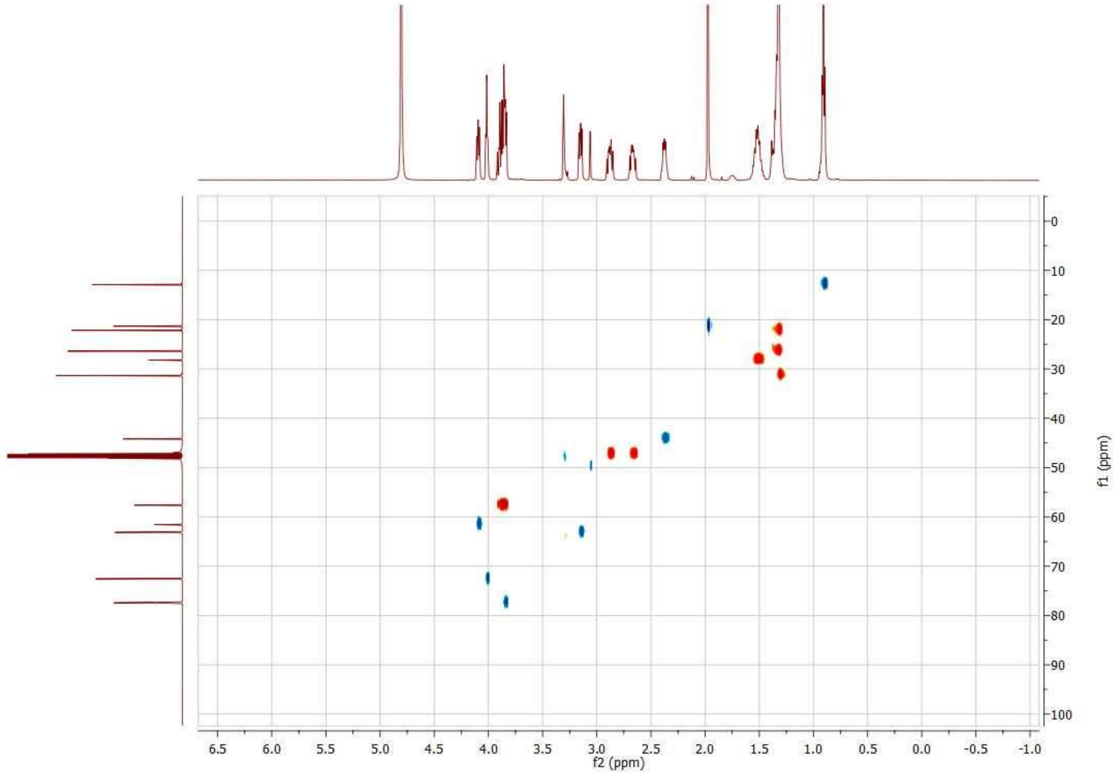

(1*S*,2*R*,3*S*,4*R*,5*R*)-*N*-(Methoxycarbonyl)pentyl-3-acetamido-4-amino-5-hydroxymethyl-cyclopentanetriol or "**2-acetamido-2-deoxy-1-(methoxycarbonylhexyl)amino-β-D-*galacto*-cyclopentane" 22**

^{1}H NMR (300 MHz, CD_3OD): compound **22**

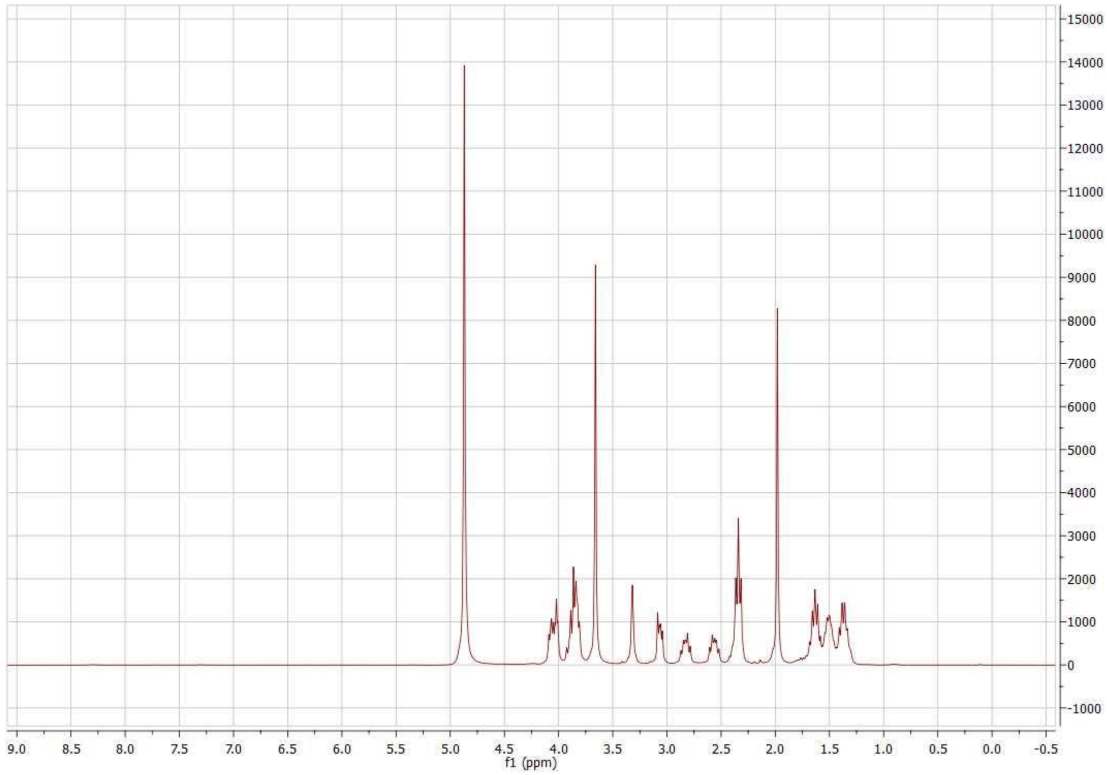

^{13}C NMR (75.5 MHz, CD_3OD): compound **22**

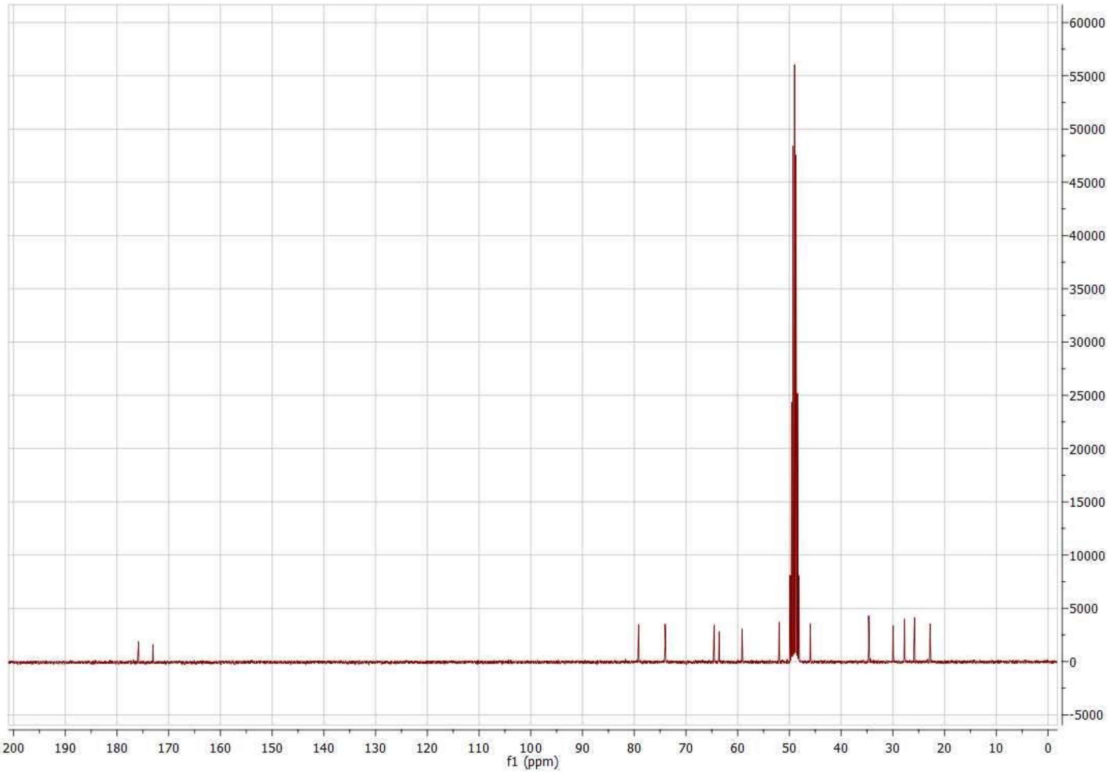

COSY (CD_3OD): compound **22**

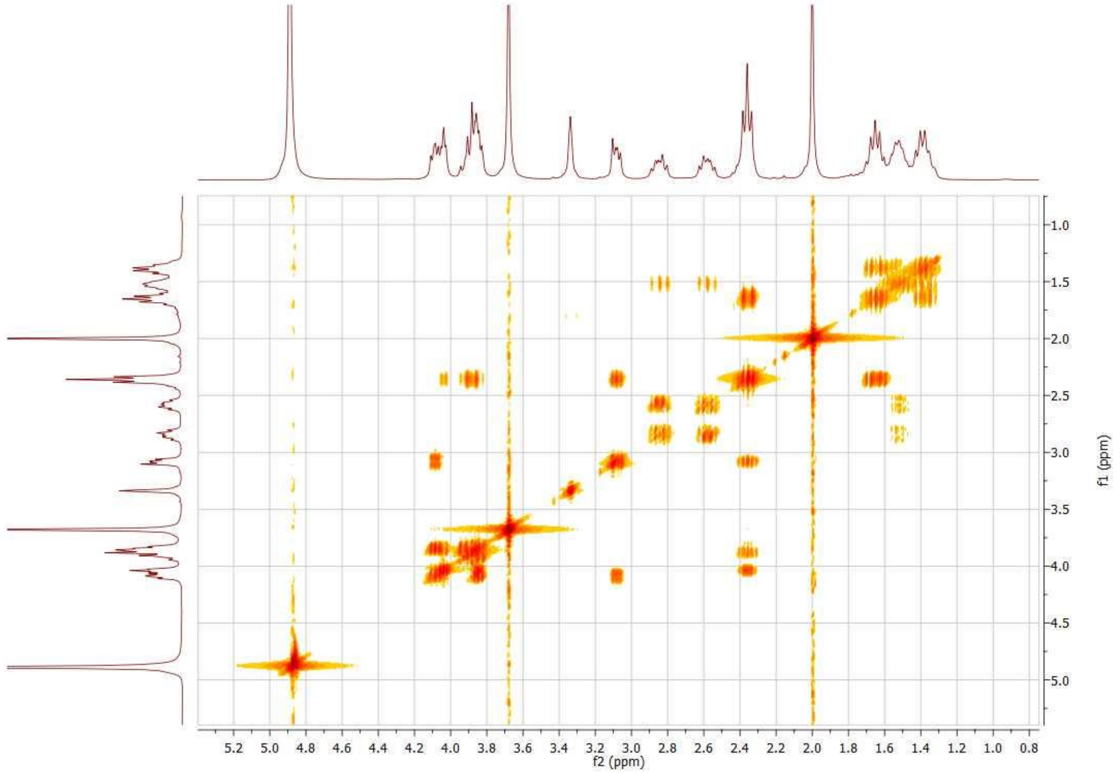

HSQC (CD_3OD): compound **22**

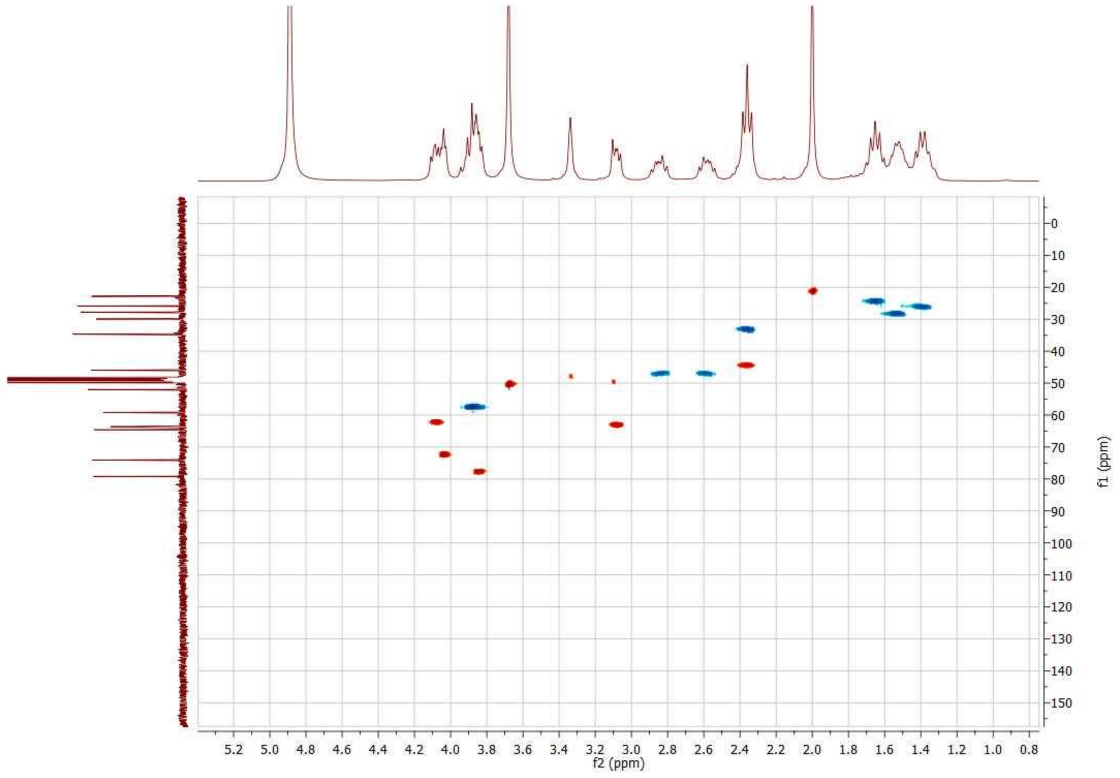

(1*S*,2*R*,3*S*,4*R*,5*R*)-*N*-(5-*C*-cyano)pentyl-3-acetamido-4-amino-5-hydroxymethyl-cyclopentanetriol or "**2-acetamido-2-deoxy-1-(5-*C*-cyano)pentylamino-β-D-*galacto*-cyclopentane" 23**

^{1}H NMR (300 MHz, CD_3OD): compound **23**

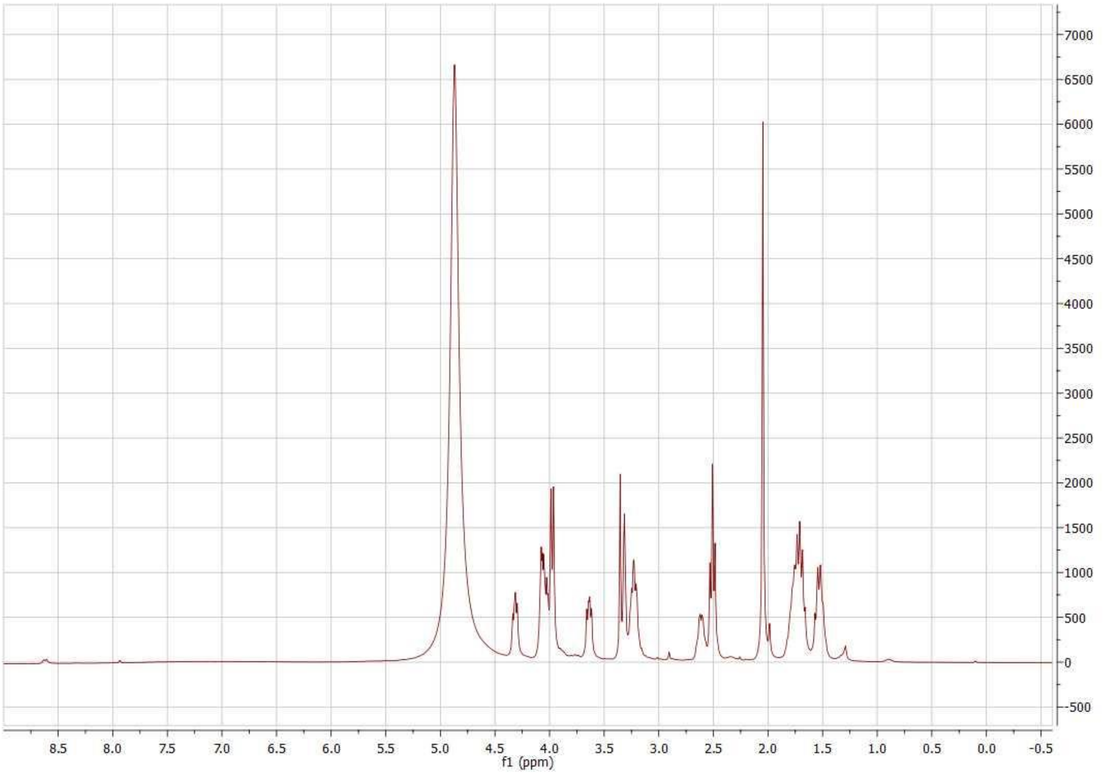

^{13}C NMR (75.5 MHz, CD_3OD): compound **23**

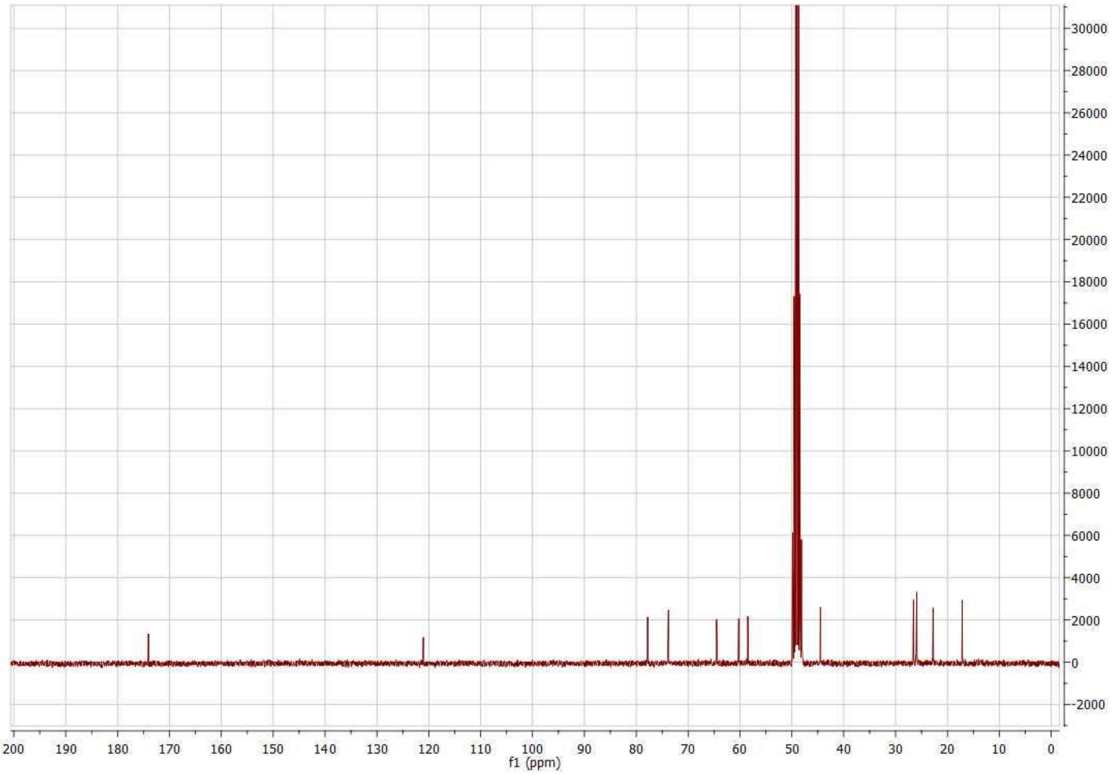

COSY (CD_3OD): compound **23**

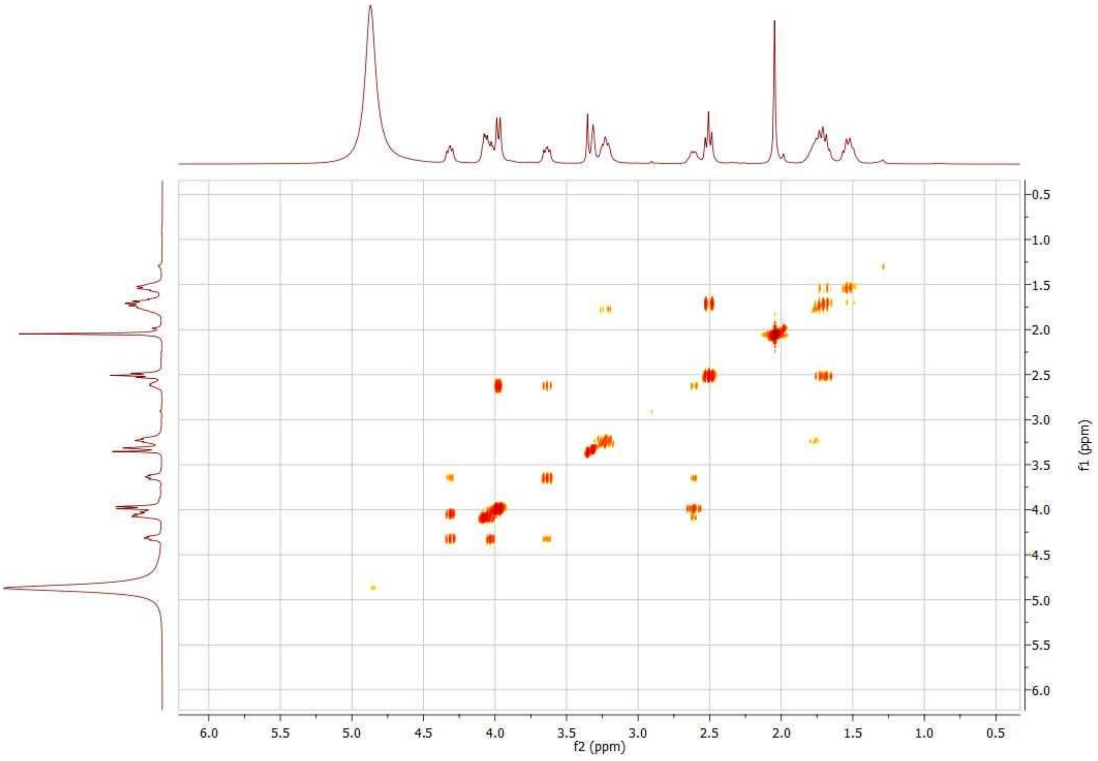

HSQC (CD_3OD): compound **23**

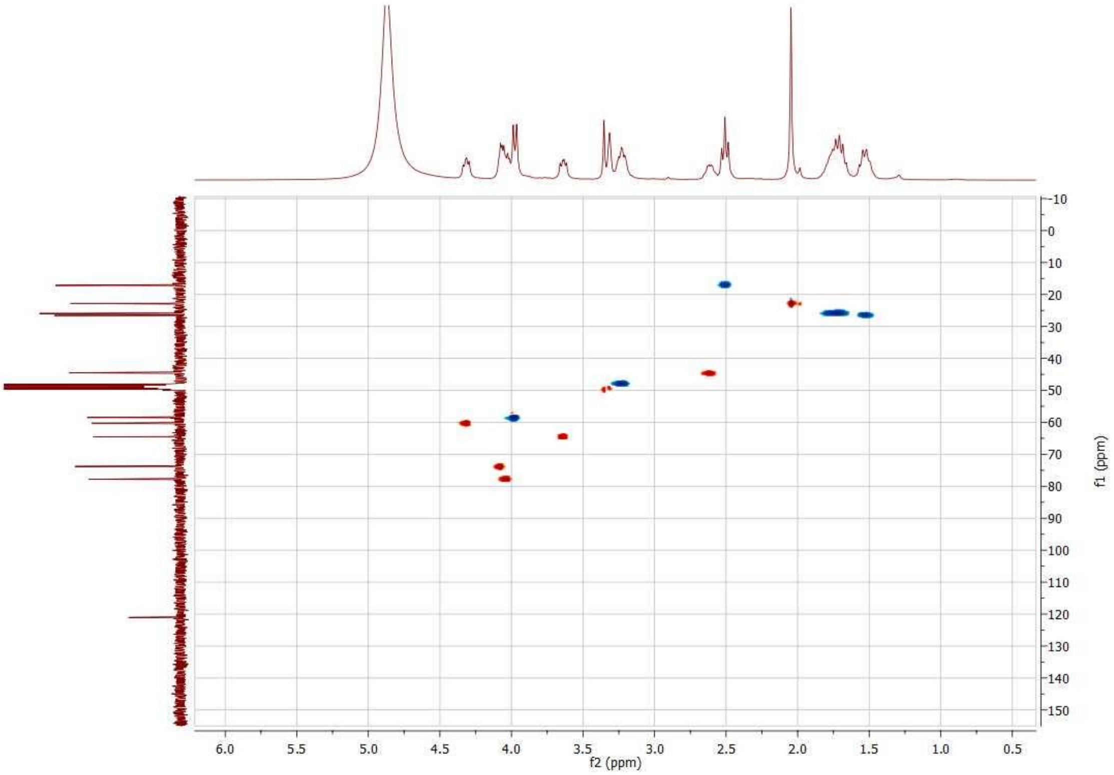

(1*S*,2*R*,3*S*,4*R*,5*R*)-*N*-(6-amino)hexyl-3-acetamido-4-amino-5-hydroxymethyl-cyclopentanetriol or “**2-acetamido-2-deoxy-1-(6-aminohexyl)amino-β-D-*galacto*-cyclopentane” 24**

^{1}H NMR (300 MHz, D_2O): compound **24**

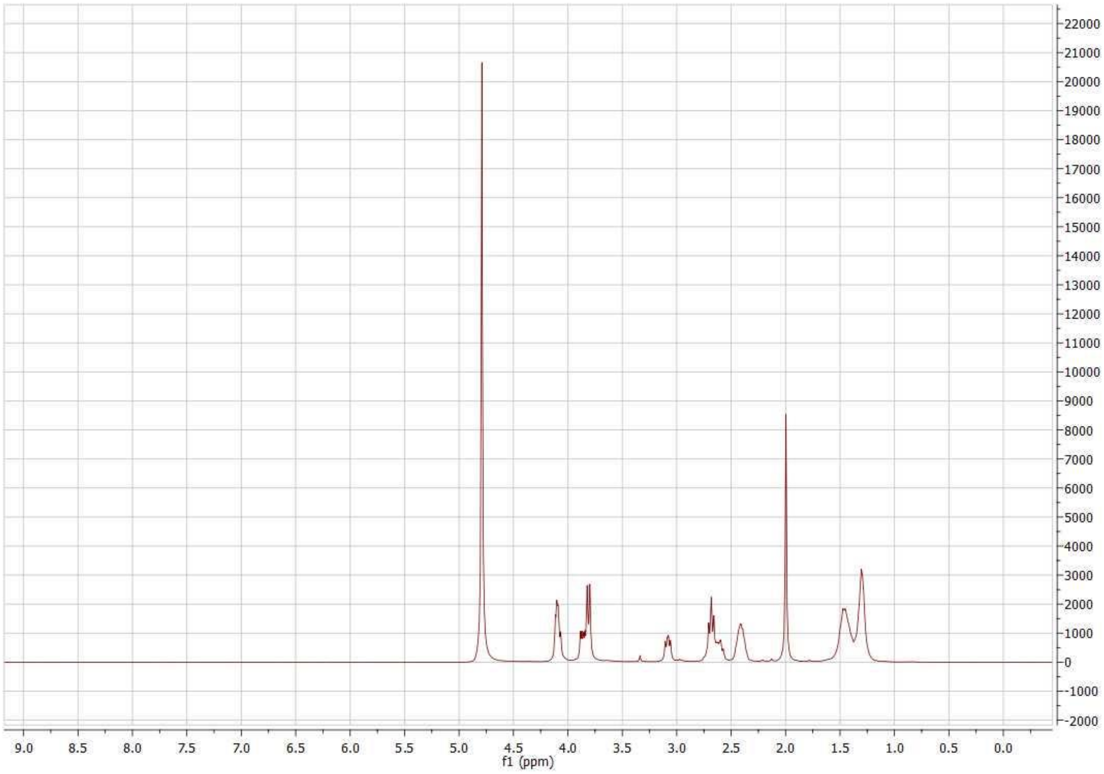

^{13}C NMR (75.5 MHz, D_2O): compound **24**

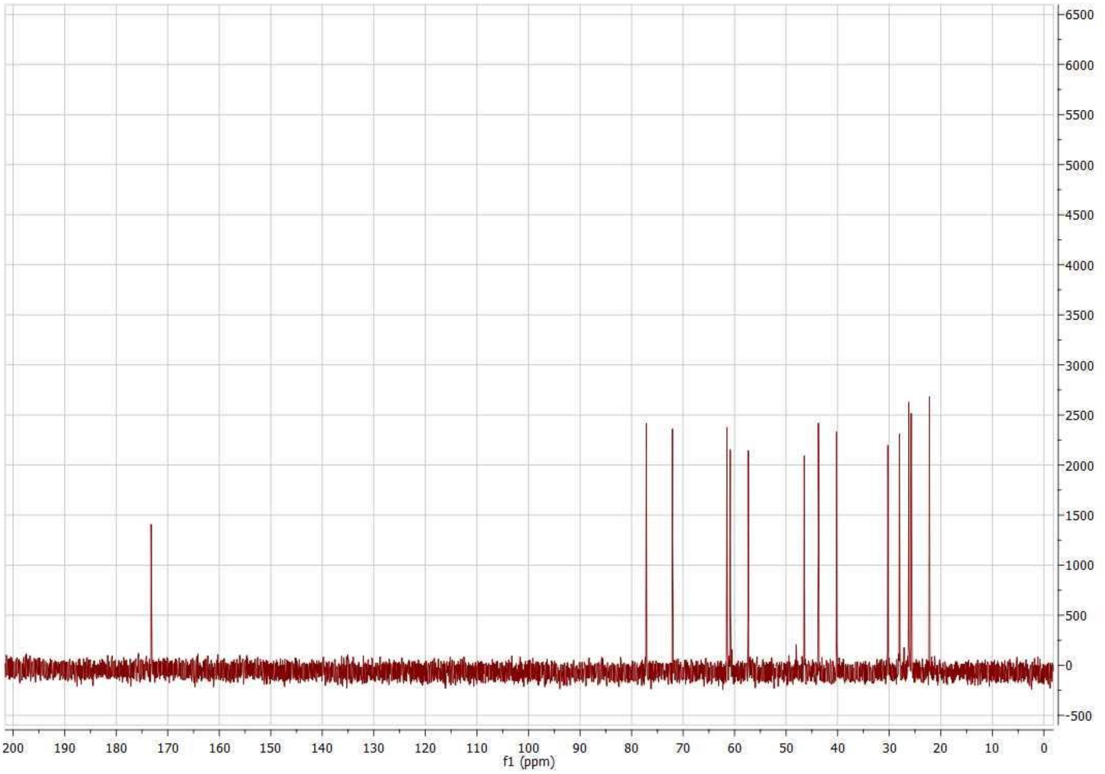

COSY (D_2O): compound **24**

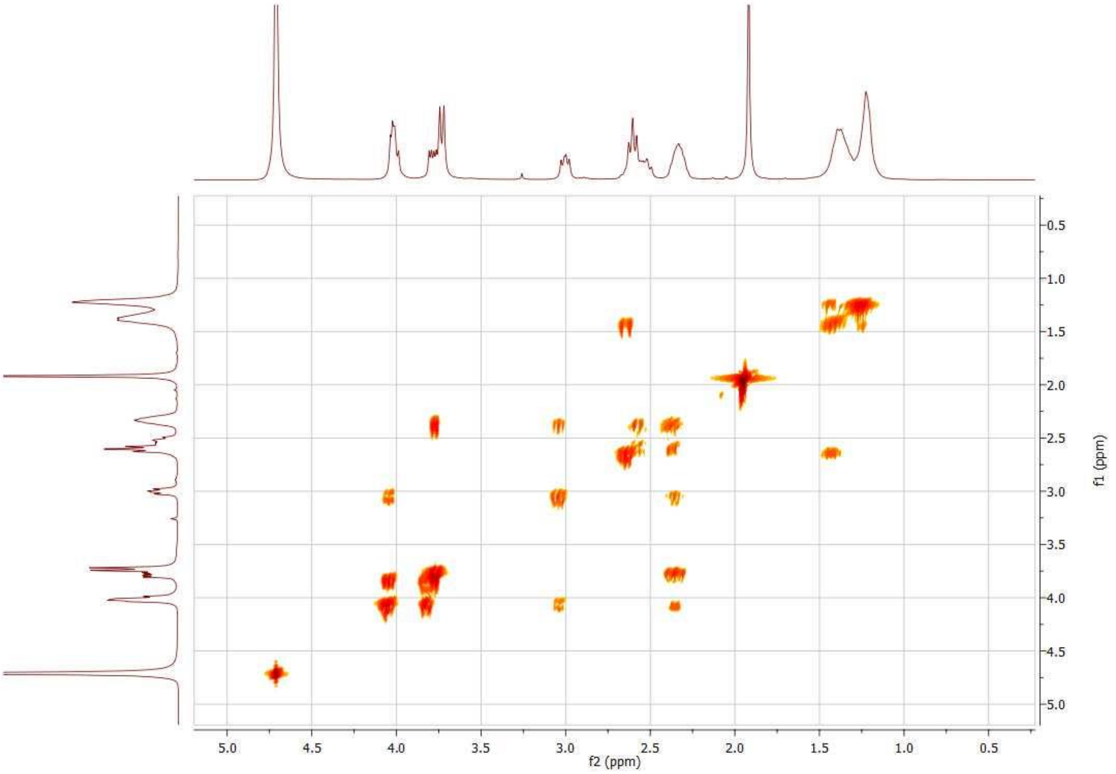

HSQC (D_2O): compound **24**

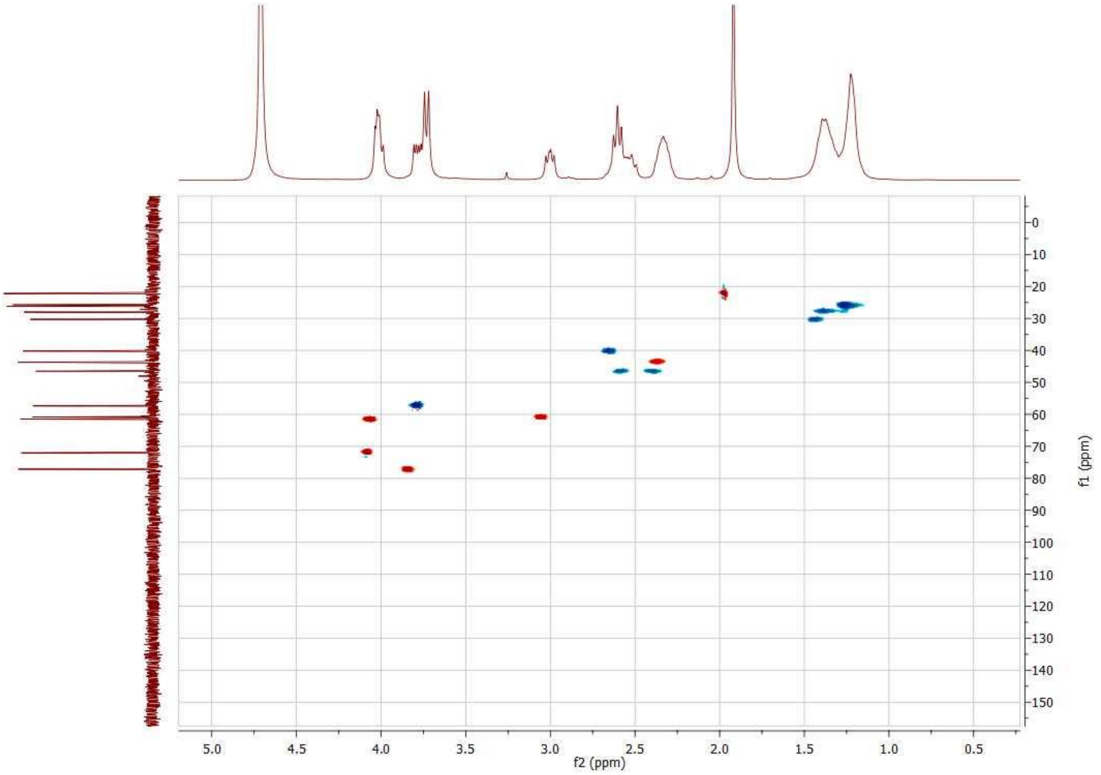

(1*S*,2*R*,3*S*,4*R*,5*R*)-*N*-(6-dansylamino)hexyl-3-acetamido-4-amino-5-hydroxymethyl-cyclopentanetriol or “**2-acetamido-2-deoxy-1-(6-dansylaminohexyl)amino-β-D-*galacto*-cyclopentane” 25**

^{1}H NMR (300 MHz, CD_3OD): compound **25**

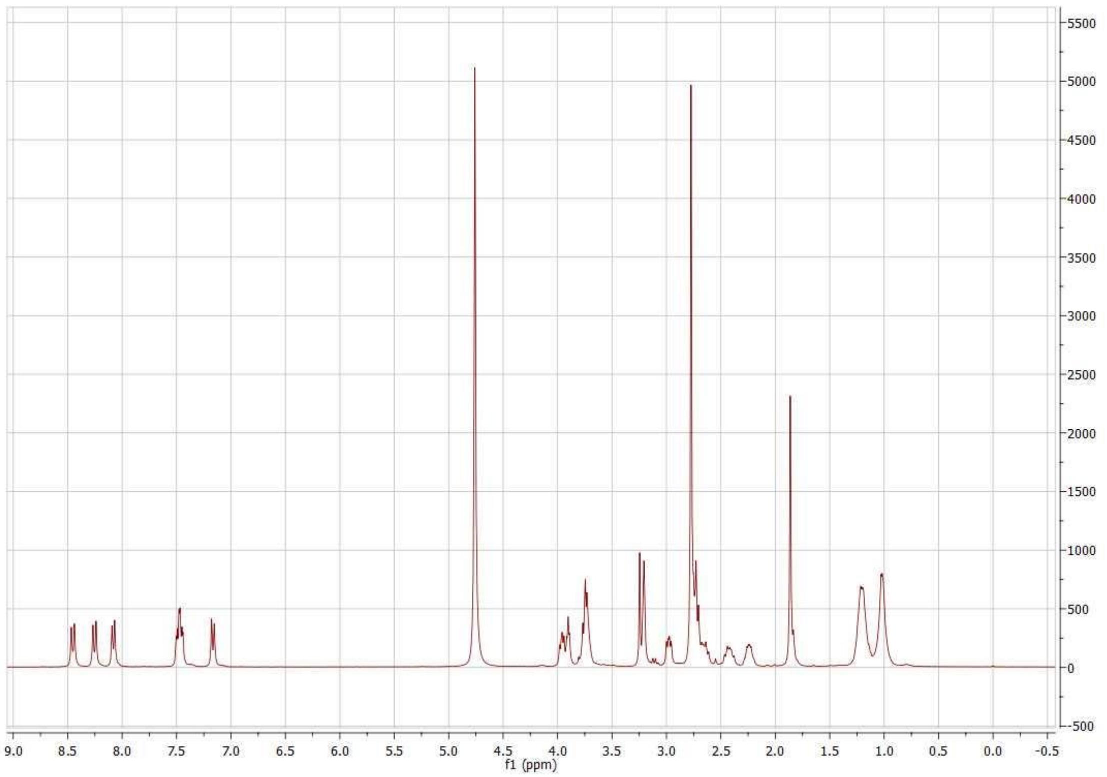

^{13}C NMR (75.5 MHz, CD_3OD): compound **25**

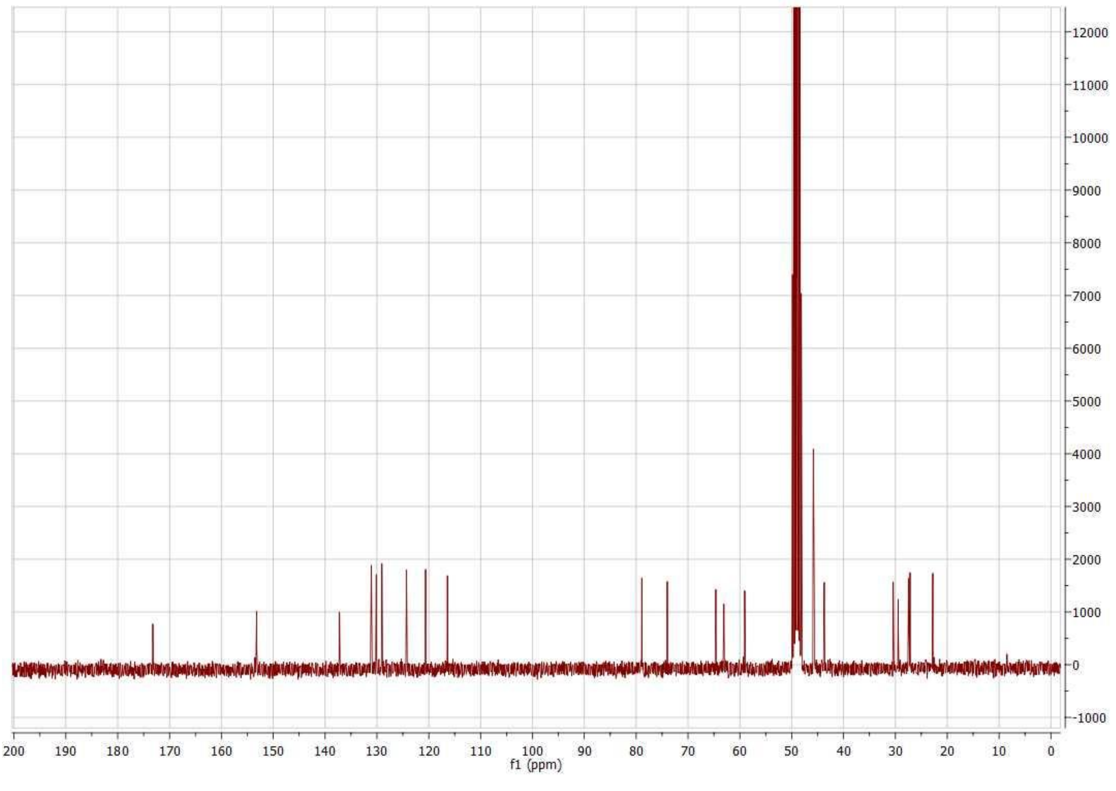

COSY (CD_3OD): compound **25**

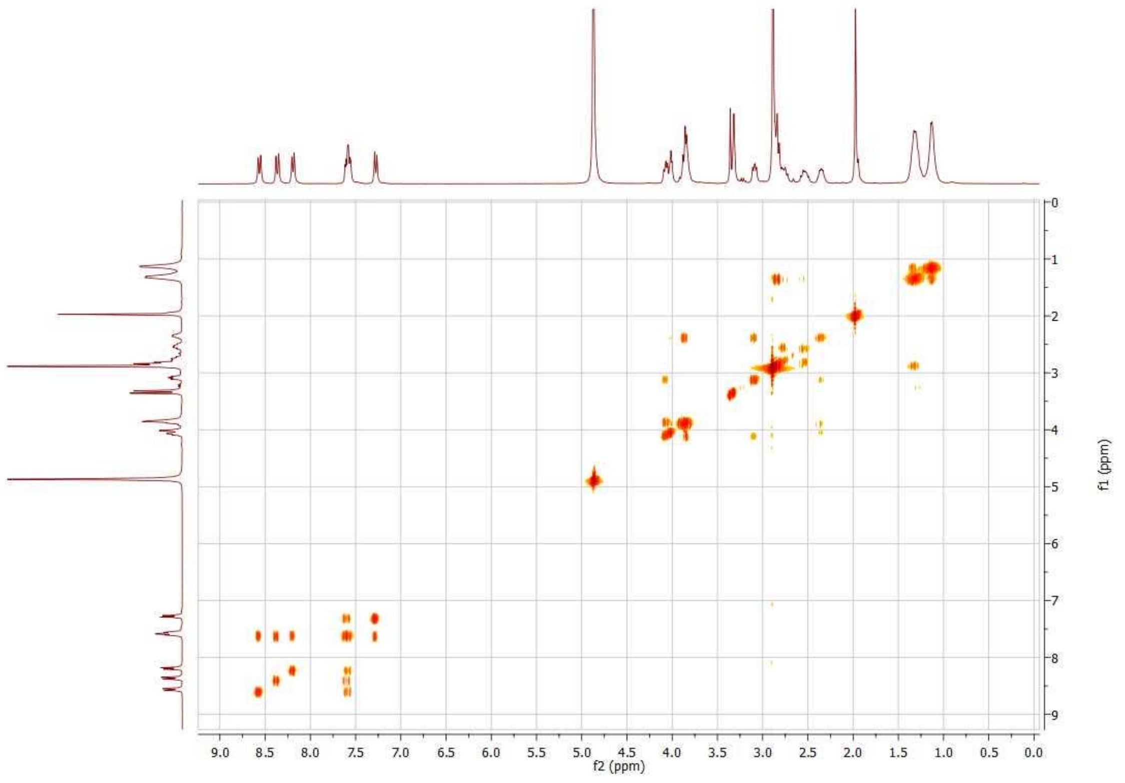

HSQC (CD_3OD): compound **25**

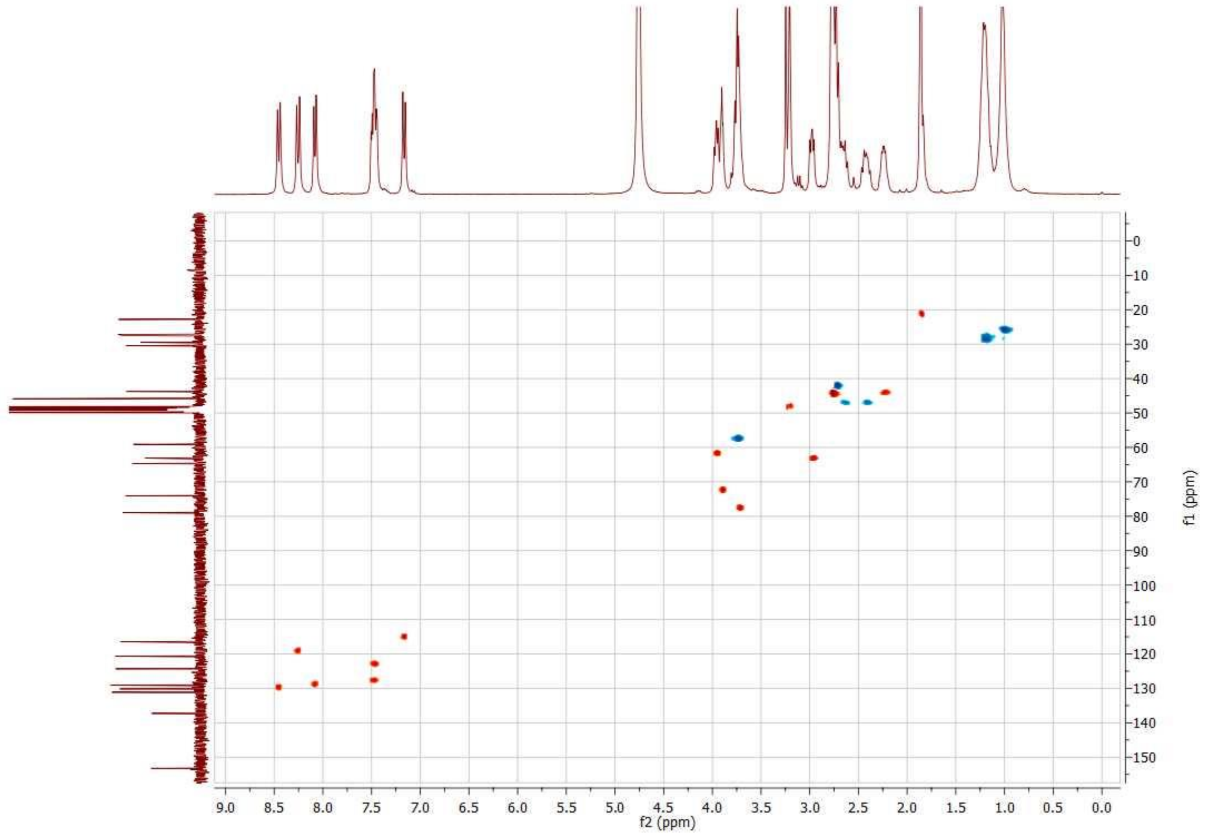

Supplement: Supplementary file 1 [file molecules-23-00708-s001.pdf]
